# Segregated input to thalamic areas that project differently to core and shell auditory cortical fields

**DOI:** 10.1016/j.isci.2024.111721

**Published:** 2025-01-01

**Authors:** Tetsufumi Ito, Mamiko Yamamoto, Li Liu, Khaleeq Ahmad Saqib, Takafumi Furuyama, Munenori Ono

**Affiliations:** 1 Systems Function and Morphology Laboratory, Graduate School of Innovative Life Science, University of Toyama, Toyama 930-0194 Japan; 2 Anatomy 2, School of Medicine, Kanazawa Medical University, Uchinada 920-0265 Japan; 3 Physiology 1, School of Medicine, Kanazawa Medical University, Uchinada 920-0265, Japan

**Keywords:** Neuroscience, Sensory neuroscience

## Abstract

Perception of the environment is multimodal in nature, with sensory systems intricately interconnected. The ability to integrate multimodal sensations while preserving the distinct characteristics of each sensory modality is crucial, and the underlying mechanisms of the organization that facilitate this process require further elucidation. In the auditory system, although the concept of core and shell pathways is well established, the brain-wide input/output relationships of thalamic regions projecting to auditory-responsive cortical areas remain insufficiently studied, particularly in relation to non-auditory structures. In this study, we utilized functional imaging and viral tracing techniques to map the brain-wide connections of core and shell pathways. We identified three distinct shell pathways, in addition to a core pathway, each exhibiting unique associations with non-auditory structures involved in behavior, emotion, and other functions. This architecture suggests that these pathways contribute differentially to various aspects of multimodal sensory integration.

## Highlights

• We identified rostral, ventral, and caudal shell auditory thalamocortical pathways
• Caudal shell thalamus receives more behavior-related input than core thalamus
• Ventral and caudal shell thalamuses are higher hierarchical order than the MGD
• The BIC is identified as the thalamic nucleus of the ventral shell pathway

## Graphical abstract

**Figure:**
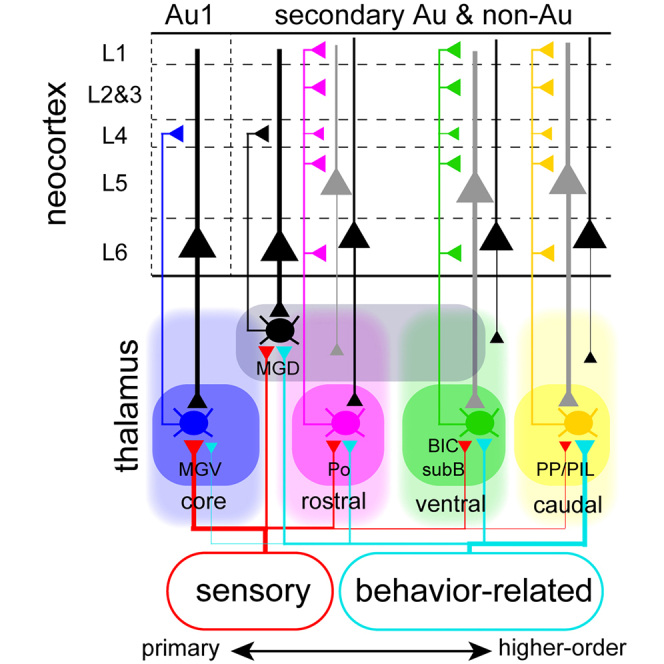

## Introduction

It is widely accepted that sensory pathways consist of core and shell divisions.^1^ Anatomically, the core cortical area is surrounded by shell areas. In the auditory system, the physiological properties of core neurons are sharply tuned to specific sound frequencies, whereas shell neurons exhibit broader tuning and multimodal responsiveness. It is generally understood that the core pathway receives predominantly auditory inputs, while the shell pathway integrates more input from non-auditory regions; however, a comprehensive brain-wide comparison between the core and shell pathways has yet to be undertaken.

In the neocortex, the auditory core and shell regions have been identified in several mammalian species.^1^ In mice, there is consensus that the core cortical field comprises two tonotopically organized regions: the anterior auditory field (AAF) and the primary auditory field (Au1).^2^^,^^3^ Additionally, other tonotopic regions have been described.^4^^,^^5^ In contrast, the precise definition of the auditory shell fields is still elusive in mice.^5^^,^^6^ For instance, the insular auditory field (IAF) demonstrates tonotopic organization^4^ but also responds robustly to somatosensory stimuli.^7^

In the thalamus, the auditory-related nuclei include the medial geniculate body (MGB) and adjacent structures. The core nucleus of the thalamus, the ventral division of the MGB (MGV), sends strong projections to layer 4 of the core cortical fields.^1^ The dorsal division of the MGB (MGD) projects predominantly to layer 4 of cortical regions adjacent to the core auditory fields.^8^^,^^9^^,^^10^ In rats, the MGV accounts for approximately 70% of the projections to tonotopic cortical fields, with the MGD providing the remainder,^11^ underscoring the importance of both structures in tonotopic auditory cortical fields. Surrounding the MGV and MGD, the suprageniculate (SG), medial division of the MGB (MGM), peripeduncular (PP), and posterior intralaminar (PIL) nuclei project more diffusely to layer 1 and deep cortical layers, not only within auditory but also within other sensory cortical areas,^12^^,^^13^ reflecting the multimodal nature of the shell domain.

Core and shell regions are similarly recognized in the auditory midbrain, the inferior colliculus (IC). The central nucleus of the inferior colliculus (ICC) projects heavily to the MGV, while the rostral, external, and dorsal cortices of the IC send projections to the surrounding thalamic regions.^14^ The external cortex of the IC, in particular, receives projections from visual^15^ and somatosensory^16^ systems, although the principal input sources for the IC cortex appear to be the same lower brainstem auditory nuclei that project to the ICC.^17^

Despite the general understanding of the connections within the core and shell pathways, the specific connections of a single pathway from input at the thalamic level to output at functionally defined auditory-responsive cortical regions have not been described in detail. A recent study^18^ utilized Cre mouse lines and Cre-dependent monosynaptic tracing to investigate the inputs and projections of shell thalamic nuclei. However, it remains unclear to what extent the input/output composition of the shell pathway diverges from that of the core pathway, particularly in relation to non-auditory regions. In this study, we employed a combination of functional imaging and pathway-specific monosynaptic tracing to investigate the input-output relationships of thalamic nuclei projecting to functionally defined regions of the auditory cortex. We performed a brain-wide analysis of the distribution of neurons providing input to these thalamic regions.

## Results

### Core and shell auditory cortical fields were identified using flavoprotein imaging

**Figure 1:**
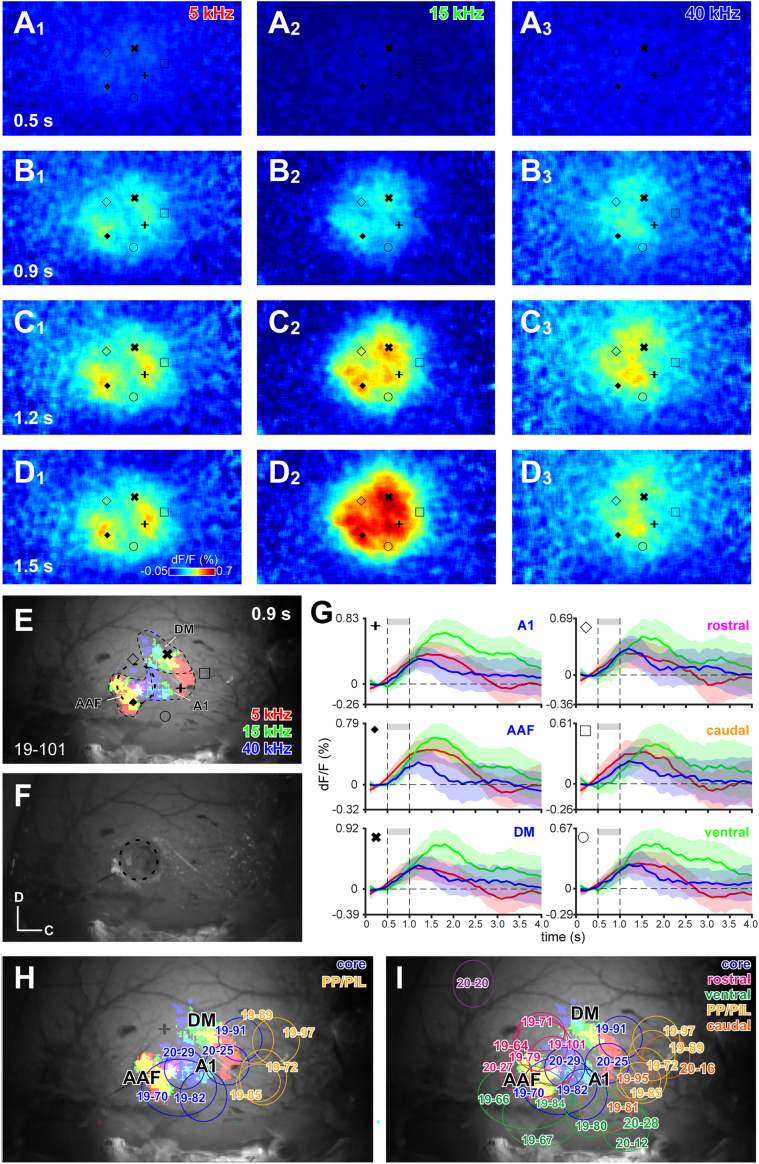
Identification of auditory cortical areas with flavin imaging (A–D) Time course of activated region in response to 5 (left), 15 (center), and 40 kHz (right) amplitude-modulated tones, which were represented from 0.5 s to 1.0 s after the start of image acquisition. Case 19–101. Note that 40 kHz activated the central part of the imaged region, whereas 5 kHz strongly activated in the rostral and caudal regions. (E) Regions of significant activation (above the threshold of 0.25% and mean – 2 SEM was higher than 0) to 5 (red), 15 (green), and 40 kHz (blue) amplitude-modulated tones at 0.9 s. Thin dotted lines indicate presumed border of AAF, A1, and DM areas. (F) After the imaging, small craniotomy (outlined with a dotted line) was made on the activated area. In this case, a hole was on the boundary of AAF (“◆”) and rostral shell (“◇”) area. (G) Time course of activity of rostral, caudal and ventral shell areas (“◇,” “□,” and “◯,” respectively), AAF (“◆”), A1 (“+”), and DM (“✖”) in response to 5 (red), 15 (green), and 40 kHz (blue) amplitude-modulated tones. The activity of shell areas was smaller than that of core areas. Solid lines indicate the average fluorescent changes. Gray shades indicate the timing of sound stimulation, and colored shades indicate 95% confidence intervals. (H and I) Summary of locations of craniotomy for injection of rgAAV-Cre. The injection sites were superimposed on an image from one case (19–101). Based on patterns of blood vessels, cranial sutures, and flavin imaging, locations of the injection site of each case were merged on a lateral view of one animal with flavin imaging (colors indicate areas with activation to 5, 15, and 40 kHz AM tones). (H) All core cases received injections into tonotopic A1, AAF, and DM areas, whereas all PP/PIL cases received injections into the caudal region. (I) Injection sites of rostral (magenta), ventral (green), caudal (yellow) and rostralmost (purple) shell cases superimposed on those of core (blue) and PP/PIL cases. The other shell-rich cases (>66% starter neurons outside the MGV) were classified into rostral, ventral, and caudal cases according to the location of injection sites. PP/PIL cases are joined into caudal cases. Note that the sites of craniotomy of core cases (blue) were almost segregated with PP/PIL main cases (pale orange), whereas in other shell cases, the sites of craniotomy were overlapped with core cases.

Using flavoprotein autofluorescence imaging,^19^ we successfully visualized cortical regions that were activated immediately following sound onset, displaying tonotopic organization (Figures 1A–1D). We identified the anterior and posterior tonotopic fields as AAF and the primary auditory field (A1; hereafter, we refer A1 as the cortical region defined by flavin imaging, whereas Au1 as the region identified with cytoarchitecture), respectively (“◆” and “+” in Figure 1). The AAF exhibited an anterior-to-posterior frequency gradient, while the A1 demonstrated a posterior-to-anterior frequency gradient (Figure 1E). In some instances, we also observed the dorsomedial (DM) tonotopic field (“✖” in Figure 1), characterized by a posteroventral-to-anterodorsal frequency gradient (Figure 1E). These tonotopic fields were classified as core auditory cortical field. The surrounding areas, which displayed weaker activation compared to the core fields (“◇,” “□,” and “◯” in Figure 1), were classified as shell auditory fields. A small craniotomy was performed on the cortical field that exhibited auditory-evoked activity (Figures 1E, 1F, 1H, and 1I). Since we injected rgAAV-Cre into the cortex beneath the site of craniotomy and performed the “tracing the relationship between input and output” (TRIO) experiment,^20^ the cortical area beneath the craniotomy site was designated as the target of the starter neurons of monosynaptic tracing.

### Starter neurons were found in various thalamic regions

**Figure 2:**
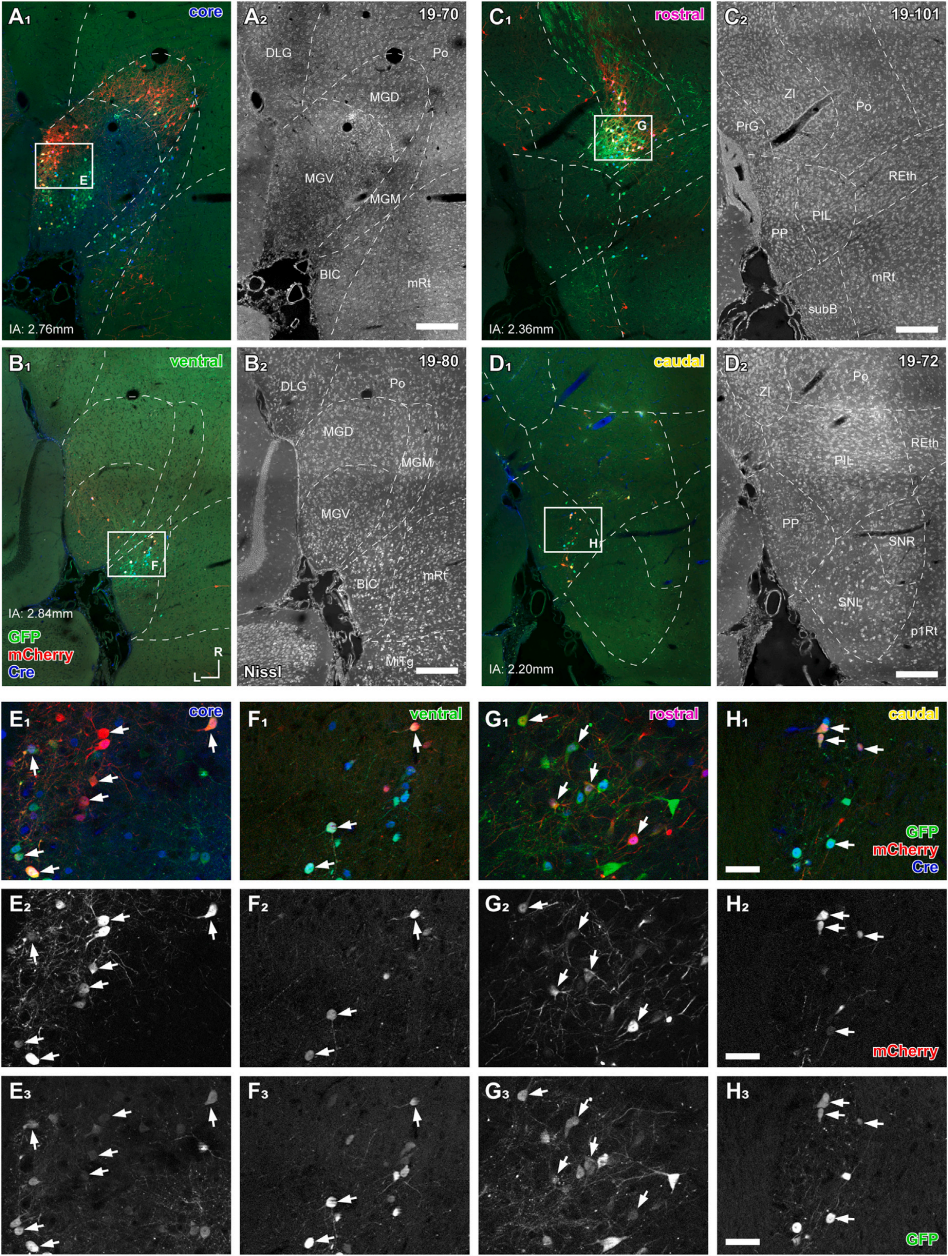
Distribution of starter neurons (A–D) Composite micrographs of horizontal sections immunostained for mCherry (red) and Cre (blue), counterstained with Neurotrace435/455 (gray). Native fluorescence of eGFP (green) was also imaged. Starter neurons were identified as cells colocalizing both eGFP and mCherry. (A) Case 19–70, where rgAAV-Cre was injected into AAF, shows Cre-positive nuclei (blue) primarily in MGD and MGV, with starter neurons mostly restricted to MGD and MGV. (B) Case 19–80, where the injection site was ventral to AAF and A1, shows starter neurons in BIC and MGV. (C) Case 19–101, where the injection site was rostral and dorsal to AAF and A1, shows starter neurons mainly in Po. (D) Case 19–72, where the injection site was caudal to A1, shows starter neurons mainly in PP and PIL. Numbers indicate the approximate dorsal distance from the interaural (IA) level. (E–H) Higher magnification views of the boxed areas in (A–D). Arrows indicate starter neurons positive for both mCherry (E_2_–H_2_) and eGFP (E_3_–H_3_). Scale bars: 200 μm (A–D) and 40 μm (E–H).

**Figure 3:**
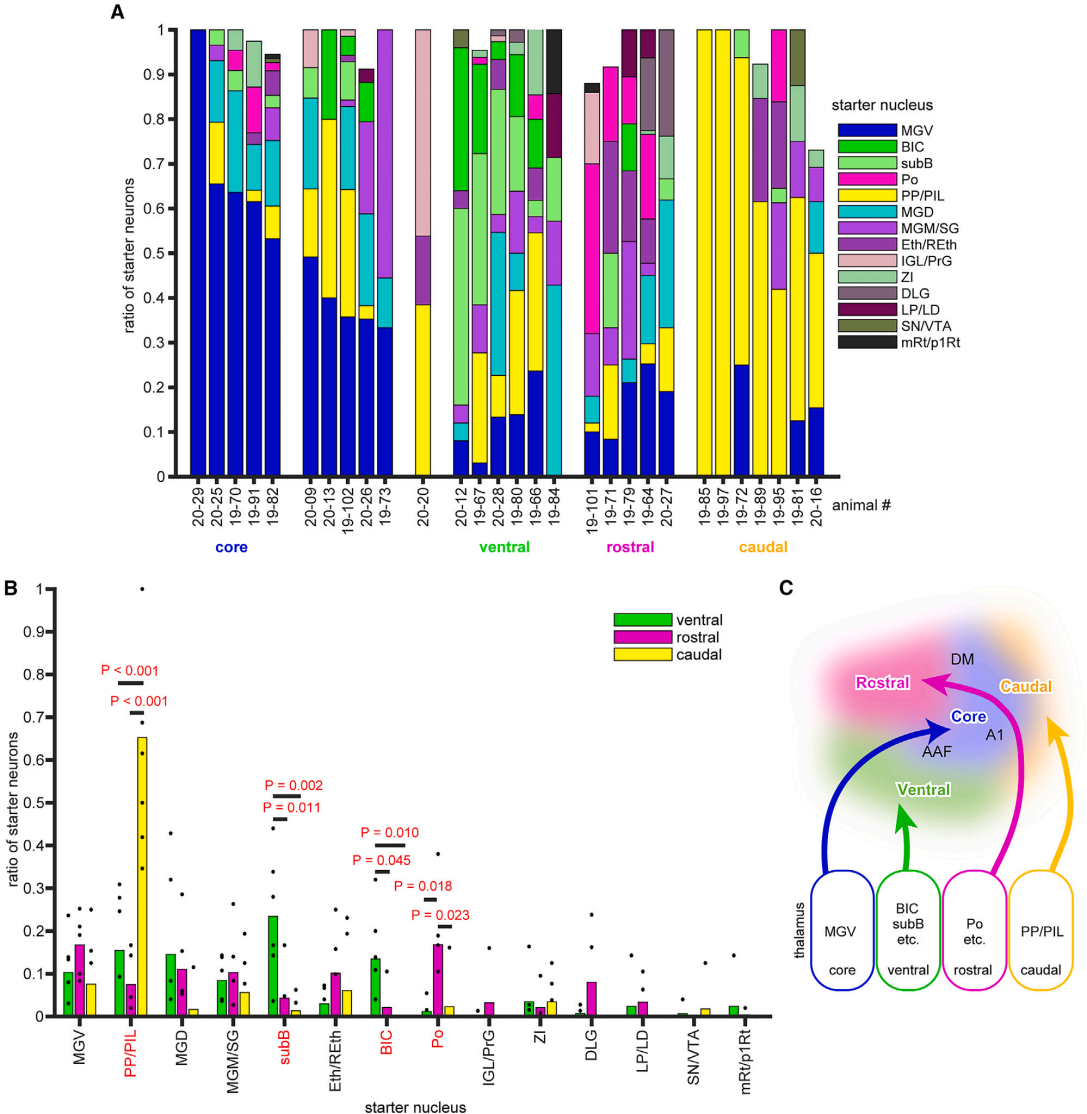
Distribution of starter neurons and relationship to rgAAV-Cre injection sites (A) The ratio of starter neurons in each starter nucleus across all cases was analyzed. Fourteen nuclei, where the maximum percentage of starter neurons exceeded 10%, were shown and analyzed. Cases where more than 50% of the starter neurons were located in the MGV were classified as “core” cases, corresponding to rgAAV-Cre injection sites in AAF and A1 (see Figures 1H and 1I). Cases where less than one-third of the starter neurons were in the MGV were classified as “shell” cases, which were further subdivided into ventral, rostral, and caudal shell cases based on the injection sites of rgAAV-Cre (see Figure 1). Among the caudal shell cases, more than 50% of the starter neurons in cases 19–72, 19–85, 19–89, and 19–97 were located in PP/PIL and were analyzed separately in Figures 5A, 8B, 8D, and 8F. (B) Distribution of starter neurons in ventral, rostral, and caudal shell cases. Dots represent individual case ratios, and bars represent the average ratio. *p* values corrected using the Tukey-Kramer method are shown. Other statistical parameters are listed in Table S2. (C) Schematic diagram summarizing significant projections based on the results.

Starter neurons, co-expressing mCherry and enhanced green fluorescent protein (eGFP), were distributed across multiple regions of the dorsal thalamus (Figures 2 and 3). According to the TRIO experiment model (Figure S1), these starter neurons are presumed to target the cortical field beneath the craniotomy site. In certain cases, the starter neurons were predominantly located in the ventral division of the medial geniculate body (MGV) (Figures 2A and 2E), while in others, they were found mainly in surrounding thalamic nuclei (Figures 2B–2D). Since tonotopic cortical fields receive dense projections from the MGV,^11^ we sorted the cases based on the ratio of starter neurons in the MGV, classifying them as core cases when the ratio exceeded 50% (Figure 3A), while the remaining cases were categorized as shell cases. All core cases (*N* = 5) involved injections into the A1 and AAF (Figure 1H). Among the shell cases, we focused on 18 instances where more than two-thirds of the starter neurons were located outside the MGV. The craniotomy sites in these shell cases surrounded those in the core cases, and we further subdivided the shell cases into rostral (*N* = 5), ventral (*N* = 6), and caudal (*N* = 7) shell cases based on the relative position of the craniotomy site on the A1 and AAF (Figure 1I).

Interestingly, the three shell categories, determined by the craniotomy site, appeared to correlate with the distribution of starter neurons: ventral shell cases exhibited a higher prevalence of starter neurons in the brachium of the inferior colliculus (BIC), located caudally to the MGB and not classified as an auditory thalamic nucleus, as well as in the subbrachial nucleus (subB) (Figures 2B, 2F, and 3). Rostral shell cases showed more starter neurons in the posterior thalamic nucleus (Po), MGM, and ethmoid thalamic nucleus (Eth), situated medially to the MGB (Figures 2C, 2G, and 3). Caudal shell cases exhibited more starter neurons in the PP and PIL nuclei (Figures 2D, 2H, and 3). In fact, in four caudal shell cases, more than 50% of the starter neurons were located in the PP/PIL region, and in some analyses, these were analyzed separately as PP/PIL cases. One unique shell case (20-20) was located farther from the A1 and AAF and did not fit into the three defined categories. This case was notable for having numerous starter neurons in the intergeniculate leaflet (IGL) and pregeniculate nucleus, located rostrally to the MGB.

Statistical analysis revealed that caudal shell cases had a significantly higher ratio of PP/PIL neurons (vs. rostral, *p* = 0.002, d = 2.99; vs. ventral, *p* = 0.007, d = 2.32). Ventral shell cases had a significantly higher ratio of BIC neurons (vs. caudal, *p* = 0.001, d = 3.36; vs. rostral, *p* = 0.013, d = 1.92) and subB neurons (vs. caudal, *p* = 0.021, d = 2.05). Rostral shell cases exhibited a significantly higher ratio of Po neurons compared to other categories (vs. caudal, *p* = 0.019, d = 2.00; vs. ventral, *p* = 0.022, d = 2.14; multiple comparison test using the Tukey-Kramer method; Figures 3B and 3C).

### Input neurons were distributed throughout various brain regions

**Figure 4:**
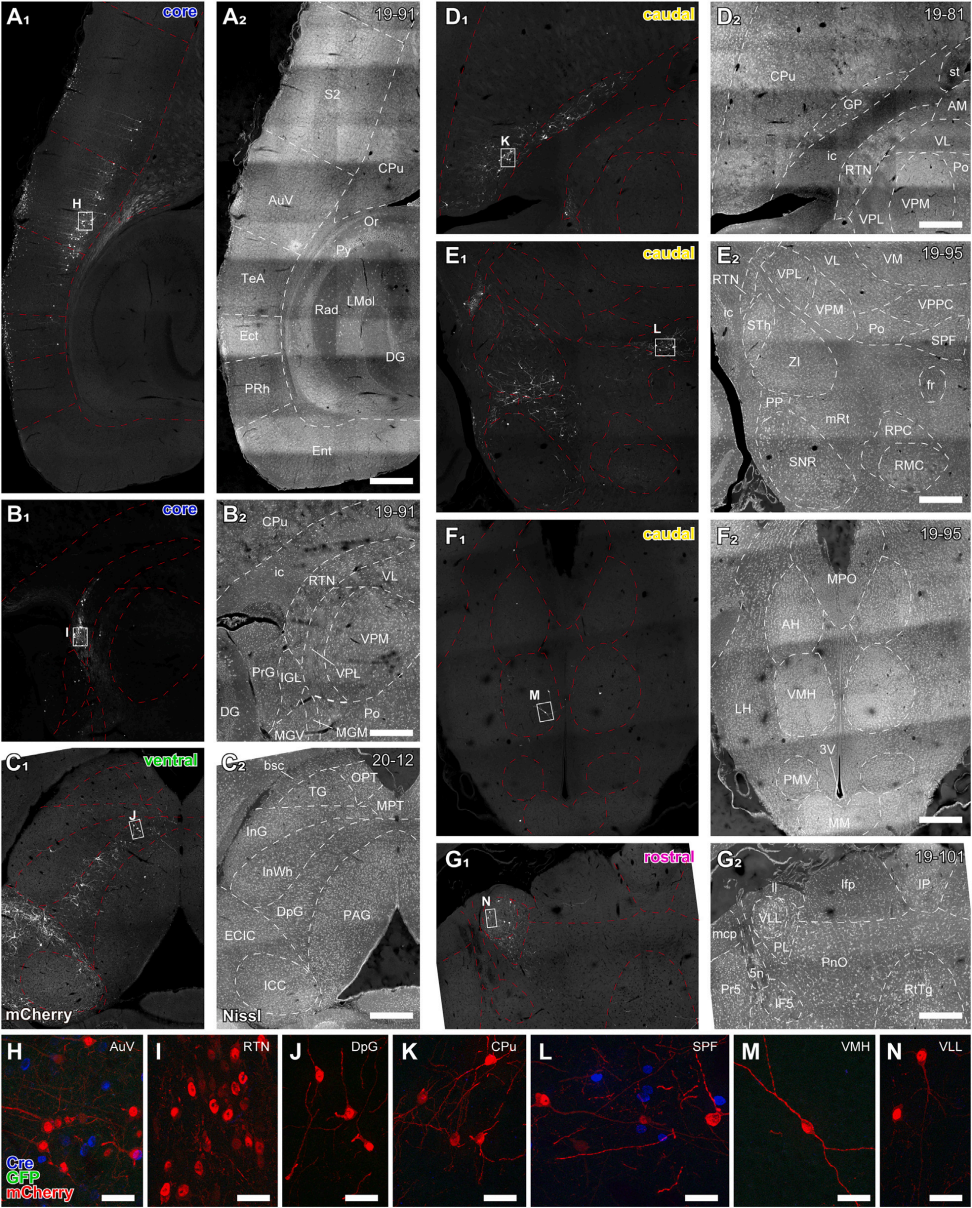
Distribution of input neurons (A–G) Composite micrographs of horizontal sections immunostained for mCherry (A_1_–G_1_), counterstained with Neurotrace435/455 fluorescent Nissl dye (A_2_–G_2_). Input neurons were identified as mCherry-positive cell bodies. (A) Neocortex of a core-targeted case (19–91). Numerous mCherry-positive pyramidal neurons are found in layer 6 of S2, AuV, TeA, and Ect. Note that AuV contains many Cre-positive cells in all layers (inset: mCherry, red; Cre, cyan), indicating that the rgAAV-Cre injection site is close to AuV. (B) In the RTN, mCherry-positive cells accumulated in the caudal part (core-targeted case 19–91). (C) In the superior colliculus, mCherry-positive cells were primarily found in the deeper layer (DpG), with fewer in the intermediate layers (InG and InWh). Few mCherry-positive cells were found in PAG (ventral-shell-targeted case 20–12). (D) In the CPu, mCherry-positive cells were mainly located in the caudal part, with few in the GP (caudal-shell-targeted case 19–81). (E) In the thalamus (caudal-shell-targeted case 19–95), mCherry-positive cells were found in RTN, ZI, SPF, PP, and mRt, with few in SNR. Note that Cre-positive cells accumulated in the SPF (inset: mCherry, red; Cre, cyan), suggesting that SPF also projects to the caudal shell region. (F) In the hypothalamus (caudal-shell-targeted case 19–95), a few mCherry-positive cells were found in AH, VMH, and LH. (G) A few mCherry-positive cells were identified in the VLL and PL of a rostral-shell-targeted case (19–101). (H–N) High-magnification, maximal-projected z stack images of the boxed regions in (A–G). Red represents mCherry; green, GFP; blue, Cre. Note that Cre-positive nuclei were only observed in AuV (near the rgAAV-Cre injection site) and SPF (which projects to the auditory cortex). No GFP-positive cell bodies were observed. Scale bars: 400 μm (A–G) and 30 μm (H–N).

Input neurons, expressing mCherry alone, were distributed across the telencephalon (Figures 4A and 4D), diencephalon (Figures 4B and 4D–4F), midbrain (Figure 4C), pons (Figure 4G), and medulla (Table S1). These neurons are considered to project to the starter neurons. In the neocortex, auditory cortical areas, somatosensory areas, visual areas, and temporal/parietal association areas on the ipsilateral side consistently contained input neurons in numerous cases (Figure 4A). The majority of input neurons in the sensory cortex were located in layer 6 (Figure 4H), although a few pyramidal neurons in layer 5 and upper layers were occasionally observed and analyzed later (cf. Figure 8). In the basal forebrain, neurons in the caudal part of the caudate-putamen (CPu) and globus pallidus (GP), which have been shown to be involved in auditory function,^21^ were consistently labeled on the ipsilateral side (Figures 4D and 4K). In the dorsal and ventral thalamus, where many mCherry-positive cells were excluded from analysis due to their proximity to starter neurons (see STAR Methods), the zona incerta (ZI), caudal part of the reticular thalamic nucleus (RTN), and subparafascicular nucleus (SPF) consistently contained input neurons on the ipsilateral side (Figures 4B–4E, 4I, 4L). In the hypothalamus, input neurons were sparsely distributed bilaterally (Figures 4F and 4M). In the midbrain, input neurons were found in the inferior colliculus (IC; ECIC and ICC), superior colliculus (SC; InG, InWh, and DpG), and periaqueductal gray (PAG) bilaterally (Figures 4C and 4J). In the pons, the nuclei of the lateral lemniscus (NLL), comprising the dorsal (DLL; Figures 4G and 4N), intermediate, and ventral nuclei (ILL, VLL), as well as the paralemniscal nucleus (PL) on the ipsilateral side, consistently contained input neurons.

A relationship between the distribution of input neurons and the target cortical areas of starter neurons was observed. For example, cases with starter neurons targeting the core auditory field contained more input neurons in the ipsilateral auditory cortex (Figure 4A) and RTN (Figure 4B). Cases with starter neurons targeting the ventral shell auditory field exhibited a greater number of input neurons in the ipsilateral IC cortex (Figure 4C). Cases with starter neurons targeting the rostral shell auditory field tended to contain more input neurons in the ipsilateral ventral complex of the lateral lemniscus (Figure 4G), RTN, and V1/V2. In cases with starter neurons targeting the caudal auditory field, more input neurons were observed in the ipsilateral basal nuclei (Figure 4D) and SPF (Figure 4E). In caudal cases, the SPF also contained Cre-positive cells (Figure 4L), which projected to the caudal shell field, indicating that the SPF projects not only to the thalamus but also to the neocortex via the same pathway. Although ZI was excluded from the analysis due to the presence of starter neurons in this region in several cases, it is noteworthy that input neurons in ZI were more prevalent in caudal shell cases.

**Figure 5:**
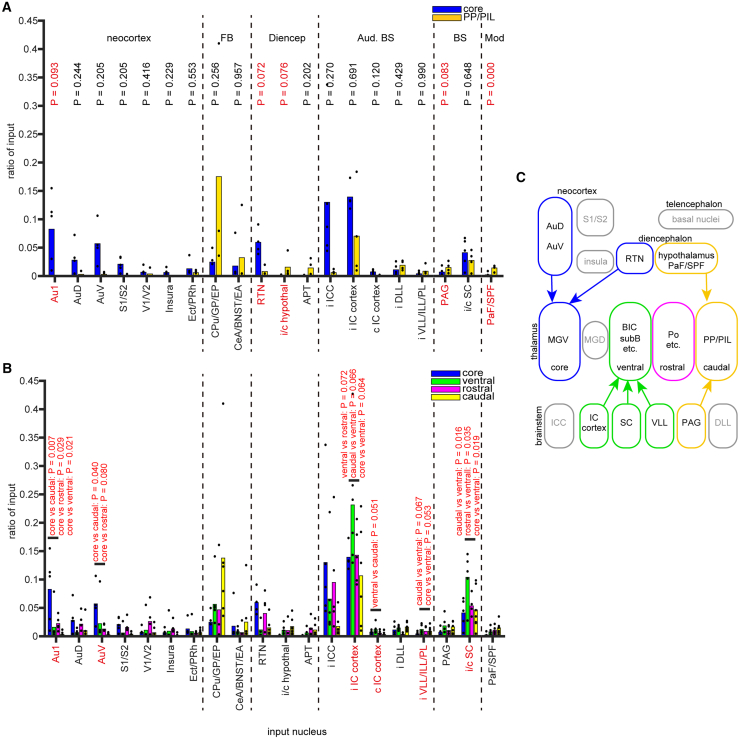
Distribution of input neurons and relationship to rgAAV-Cre injection sites (A) The ratio of input neurons in core-targeted cases (*N* = 5) and PP/PIL-targeted cases (*N* = 4) across different brain regions: neocortex, forebrain (FB), diencephalon (Diencep), auditory brainstem (Aud. BS), other brainstem (BS), and modulatory (Mod) areas. Twenty nuclei with an average percentage of input neurons above 0.5% and a maximum percentage above 1% were included in the analysis. Dots represent individual case ratios, and bars represent the average ratio. The mean ratios were compared, and *p* values corrected using the Tukey-Kramer method are shown. Other statistical parameters are listed in Table S3. (B) The ratio of input neurons across brain areas in core-targeted cases (*N* = 5), ventral-shell-targeted cases (*N* = 6), rostral-shell-targeted cases (*N* = 5), and caudal-shell-targeted cases (*N* = 7). Dots represent individual case ratios, and bars represent the average ratio. *p* values corrected using the Tukey-Kramer method are displayed if lower than 0.100. Other statistical parameters are listed in Table S4. (C) Schematic diagram summarizing significant projections based on the results. Gray areas represent brain regions with no significant relationships, though they are included in later figures.

We grouped neighboring nuclei with similar functions or cytoarchitecture and excluded input nuclei where the mean input ratio was less than 0.5% and the maximal input ratio was less than 1% from the analysis. While input from the cochlear nucleus and superior olivary complex was consistently observed, the number of neurons was small and, thus, excluded from further analysis. Finally, twenty brain regions remained for further analyses (Figures 5A and 5B).

We compared the ratio of input neurons in each nucleus between groups defined by the cortical targets of the starter neurons. To assess clearer trends, we focused on cases where starter neurons were clustered in specific nuclei, rather than those with mixed populations of starter neurons. We compared the core group (*N* = 5) and four cases (19–72, 19–85, 19–89, and 19–97) from the caudal group, where more than 50% of starter neurons were located in PP/PIL, and found that core cases had significantly fewer input neurons in PaF/SPF compared to PP/PIL cases (*p* < 0.001, d = 2.61). Furthermore, core cases tended to have more input neurons in the ipsilateral Au1 and RTN, and fewer input neurons in the hypothalamus and PAG compared to PP/PIL cases (*p* = 0.093, 0.072, 0.076, and 0.083, d = 1.80, 2.77, −1.13, and −2.61, respectively; multiple comparison test using the Tukey-Kramer method; Figure 5A). Next, we performed multiple comparisons among the core, ventral, rostral, and caudal shell groups. Core cases contained more input neurons in Au1 than the rostral, ventral, and caudal shell cases (*p* = 0.029, 0.021, and 0.007, d = 1.38, 1.44, and 2.06, respectively), and more input neurons in AuV than the rostral and caudal shell cases (*p* = 0.080 and 0.040, d = 1.66 and 2.32, respectively; multiple comparison test using the Tukey-Kramer method; Figure 5B). Ventral shell cases tended to have more input neurons in the ipsilateral IC cortex compared to other pathways (0.050 < *p* < 0.100), and more input neurons in the contralateral IC cortex compared to the rostral pathway (*p* = 0.051, d = 1.37). The ratio of input neurons in the ipsilateral VLL/ILL/PL tended to be higher in ventral cases compared to core and caudal shell cases (*p* = 0.053 and 0.067, d = 1.16 and 0.37, respectively; multiple comparison test using the Tukey-Kramer method; Figure 5B).

These findings suggest that the composition of input neurons differs depending on the cortical targets of starter neurons. Based on these results, we propose that the auditory pathway to the cortex comprises one core pathway and three shell pathways, each characterized by distinct combinations of thalamic neurons, their presynaptic inputs, and postsynaptic cortical targets (Figure 5C).

### The composition of IC projection cell types differs between thalamic pathways

**Figure 6:**
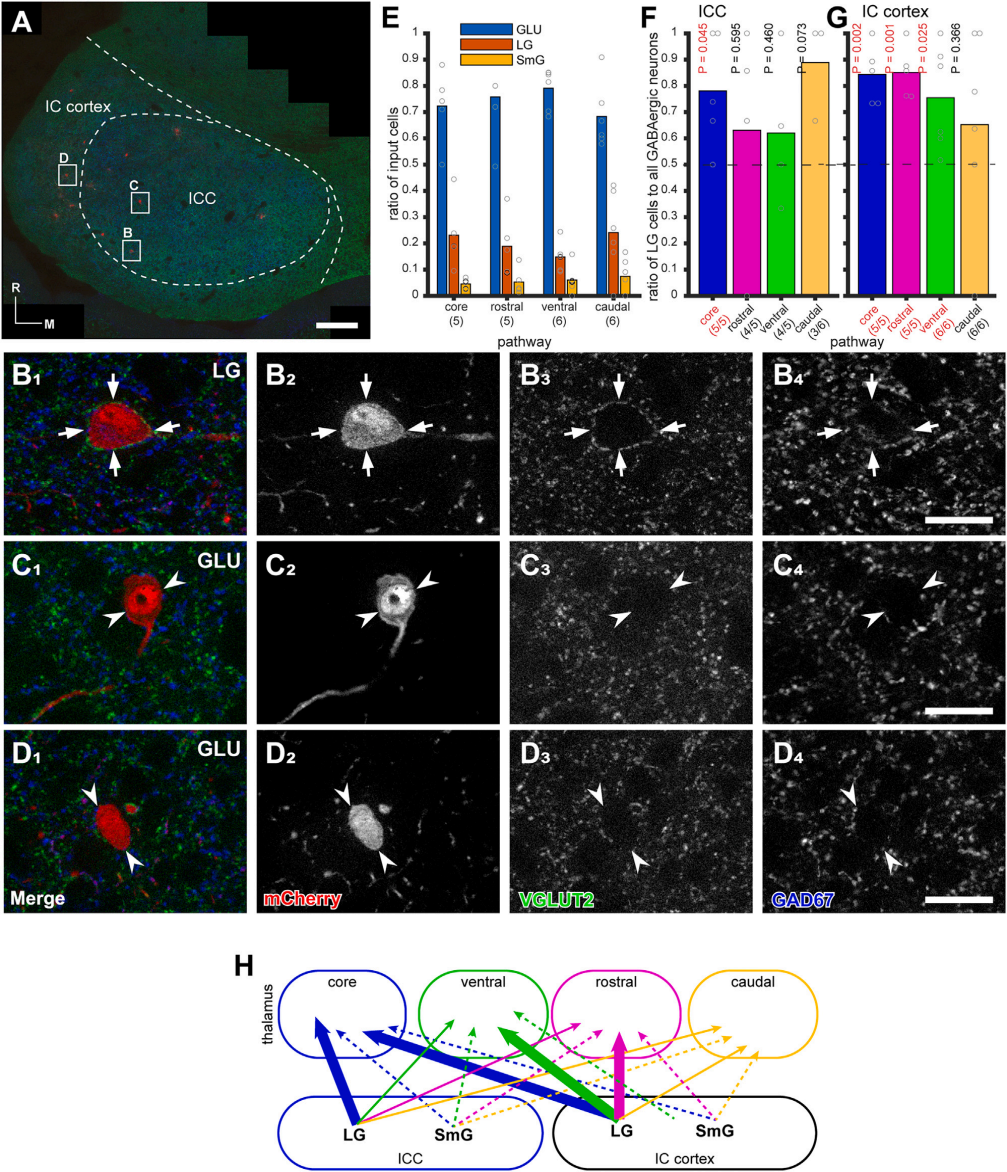
Immunohistochemical identification of IC cell types projecting to the thalamus (A) A composite micrograph of a horizontal section of IC immunostained for VGLUT2 (green), mCherry (red), and GAD67 (blue). Case 20–29. mCherry-positive input neurons are distributed in both the ICC and the IC cortex on the ipsilateral side of the injection site. (B–D) Large GABAergic (LG, arrows) and putative glutamatergic (GLU, arrowheads) neurons were identified. GAD67-positive somatic labeling was present in LG neurons (B) but absent in GLU neurons (C and D). Scale bars: 200 μm (A) and 30 μm (B–D). (E) The ratio of GLU, LG, and small GABAergic (SmG) input neurons across cases classified by the rgAAV-Cre injection site. Dots represent individual case ratios, and bars represent the average ratio. The ratios of each cell type remain similar, regardless of classification. Numbers in parentheses indicate the number of animals. (F and G) The ratio of LG cells to all GABAergic input cells in the ICC (F) and IC cortex (G) across cases classified by the rgAAV-Cre injection site. Dots represent individual case ratios, and bars represent the average ratio. A one-sample t test was used to determine whether the mean ratio significantly differed from 0.5, revealing that LG cell ratios were significantly higher than 0.5 in several pathways, suggesting that the contributions of different GABAergic cell types vary among pathways. The numerator and denominator in parentheses indicate the number of animals containing GABAergic input neurons and the total number of animals, respectively. Other statistical parameters are listed in Table S5. (H) Schematic diagram of GABAergic projections from the IC. Solid lines represent projections from LG neurons, and dotted lines represent projections from SmG neurons. The thickness of the lines indicates the relative strength of the projection.

As most inputs from auditory brainstem nuclei converge in the IC, the IC serves as the primary source of ascending auditory information to the thalamic regions. Given this, it is crucial to analyze the composition of inputs from the IC to each thalamic pathway in detail. To achieve this, we identified three types of IC neurons—large GABAergic (LG), small GABAergic (SmG), and glutamatergic (GLU) neurons—each characterized by distinct neuronal circuitry^22^^,^^23^^,^^24^^,^^25^^,^^26^ and different sound responsiveness.^27^^,^^28^^,^^29^ These neurons were identified among the mCherry-positive neurons in the IC (LG neurons: arrows; GLU neurons: arrowheads; Figures 6A–6D).

In general, all three cell types projected to the thalamic regions of the four pathways. Approximately 70% of the tectothalamic projections consisted of GLU neurons, 20% consisted of LG neurons, and the remainder were SmG neurons (Figure 6E). However, the composition of GABAergic projections from the IC appeared to vary across pathways. We therefore analyzed the ratio of LG neurons to total GABAergic neurons projecting to the thalamus in each pathway. In the core pathway, LG neurons represented the majority of GABAergic tectothalamic projection neurons in both the ICC (*p* = 0.045, d = 1.29, paired t test; Figure 6F) and the IC cortex (*p* = 0.002, d = 3.03, paired t test; Figure 6G). Additionally, in the rostral and ventral pathways, LG neurons constituted the majority of GABAergic tectothalamic projection neurons in the IC cortex (*p* = 0.001 and 0.025, d = 3.54 and 1.28, paired t test; Figure 6G). These findings suggest that LG neurons in the ICC play a critical role in the core auditory pathway, while those in the IC cortex are likely important for the core, ventral, and rostral shell pathways (Figure 6H).

### Pathway-specific composition of input neurons revealed by correlation analysis

As demonstrated by the previous analyses, thalamic neurons in different pathways appear to receive inputs from distinct combinations of nuclei. For instance, the MGV receives more input from Au1, AuV, and RTN compared to the caudal shell thalamus (Figure 5B). This suggests that the presence of input neurons in Au1 is likely associated with the presence of input neurons in AuV and RTN, indicating a positive correlation between the input ratios of these regions. In general, clusters of positive correlations between input nuclei indicate functional connectivity.

**Figure 7:**
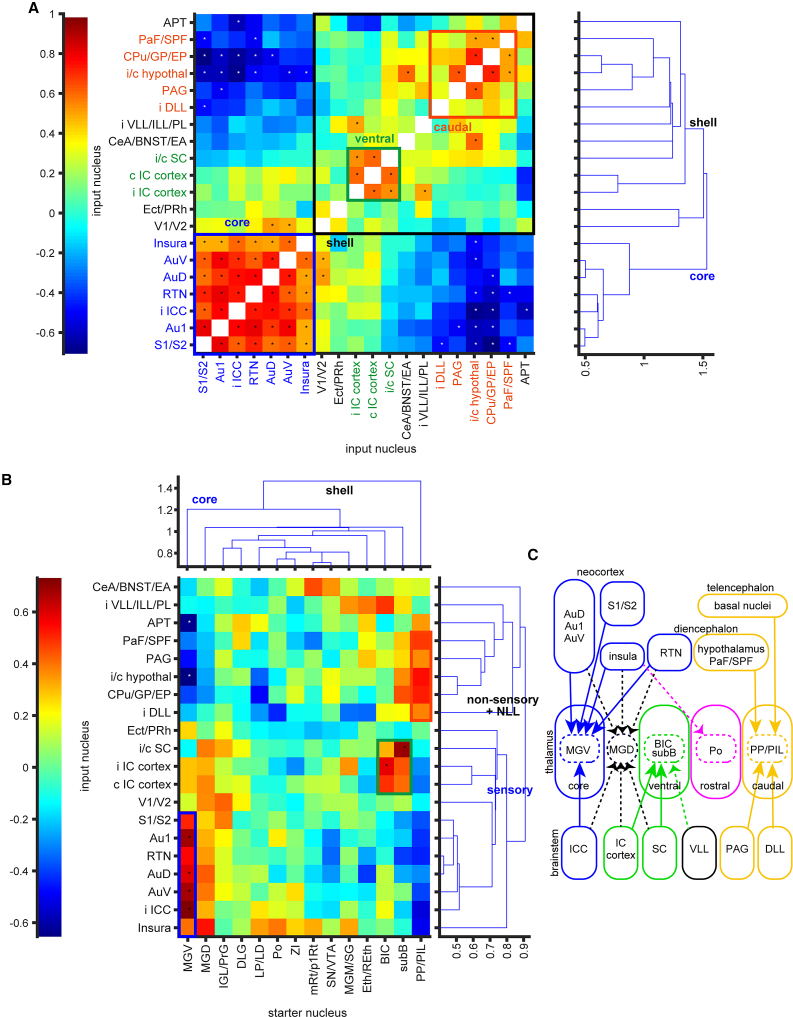
Correlation analysis (A) Correlation of input ratios among brain areas. The color scale represents the correlation coefficient, with asterisks indicating statistical significance (*p* < 0.05 after BH-FDR correction). From the cluster analysis (right), input nuclei were categorized into core and shell domains. Based on the starter-input correlation shown in (B), shell domains were further subdivided into ventral and caudal shell domains. (B) Correlation of input and starter ratios among brain areas. The orange box highlights the relationship between PP/PIL and several input nuclei, suggesting that these input nuclei are associated with the caudal shell domain. The green box shows the relationship between subB/BIC and several input nuclei, particularly SC and the IC cortex, indicating that these input nuclei are associated with the ventral shell domain. The blue box demonstrates the relationship between MGV and several input nuclei, particularly with the auditory cortex, indicating that these input nuclei are related to the core domain. (C) Schematic diagram based on the correlation analysis. Solid lines indicate projections with strong positive correlations, while dotted lines represent weaker positive correlations connecting different pathways. Statistical parameters related to this Figure are listed in Tables S6–S9.

To investigate this, we calculated Spearman’s correlation coefficients for the input neuron ratios between all combinations of 20 nuclei (Figure 7A). Cluster analysis of the correlation coefficients (right panel in Figure 7A) revealed two distinct clusters of input nuclei. One cluster, which included auditory core regions such as the ipsilateral ICC and Au1, was classified as the core cluster. The other cluster, which comprised subcortical non-auditory nuclei, was classified as the shell cluster. The core cluster also included other sensory cortical areas (S1/S2 and insular cortex) and the RTN. While V1/V2 were not included in the core cluster, they showed correlations with certain nuclei within the cluster. All nuclei in the core cluster were positively correlated with one another and negatively correlated with some nuclei in the shell cluster. Although the shell cluster was not as clearly defined as the core cluster, it appeared to be further subdivided into two subclusters: one containing the IC cortex and SC, and the other containing the DLL, PAG, hypothalamus, basal nuclei (CPu/GP/EP), and PaF/SPF. The ventral lateral lemniscus nuclei (VLL/ILL/PL) and amygdala (CeA/BNST/EA) were included in the shell cluster but were not clearly separated between the two subclusters.

The presence of more input neurons in Au1 suggests that starter neurons are more likely to be located in the MGV rather than in shell thalamic nuclei (Figure 4). Thus, the relationship between clusters of input neurons and starter nuclei is detectable as a positive correlation between input and starter neuron ratios. To explore this, we calculated Spearman’s correlation coefficients between the ratios of input neurons in the input nuclei and the ratios of starter neurons in the starter nuclei (Figure 7B). As expected, input nuclei classified as part of the core cluster showed a high correlation with the MGV as the starter nucleus. Similarly, one shell subcluster showed a high correlation with subB and BIC, while the other shell subcluster correlated with PP/PIL. Cluster analysis of starter nuclei classified MGV and PP/PIL into both extremes, and other nuclei in the center, suggesting hierarchy of shell nuclei. Cluster analysis of input nuclei revealed two clusters, one containing sensory regions, and another containing mostly non-sensory regions. Based on these relationships, we classified the subcluster associated with BIC and subB as the ventral shell subcluster, and the subcluster associated with PP/PIL as the caudal shell subcluster. The rostral shell thalamic nucleus Po displayed a weak correlation with the insular cortex, along with LP/LD and MGM/SG. Interestingly, the MGD showed weak positive correlations with both the core cluster (e.g., Au1) and the ventral shell cluster (e.g., SC).

These results suggest that the core, ventral, and caudal shell pathways are segregated based on input to the thalamus, with MGD functioning as a hub between the core and ventral pathways (Figure 7C).

### Cortical regions and layers that give rise to corticothalamic projections differ among pathways

**Figure 8:**
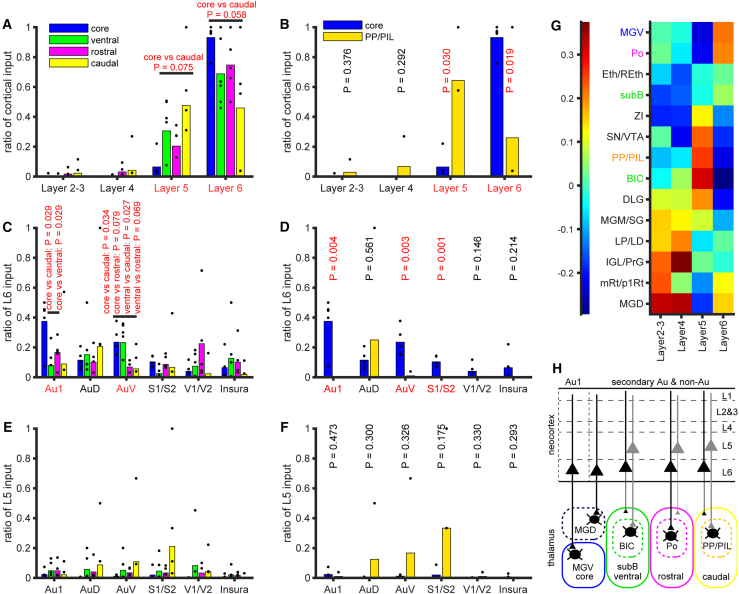
Layer distribution of cortical input neurons (A) The ratio of input from each cortical layer to all cortical input in the four pathways. Dots represent individual case ratios, and bars represent the average ratio (same applied to B–F). The core thalamus tended to receive more input from layer 6 compared to the caudal shell thalamus. (B) The ratio of input from each cortical layer to all cortical input in MGV and PP/PIL. MGV received significantly more input from layer 6 and less input from layer 5 than PP/PIL. (C) The ratio of layer 6 input neurons to all cortical input neurons in the four pathways. The core thalamus received more cortical input neurons from Au1 layer 6 than the caudal and ventral shell thalamus, and from AuV layer 6 than the caudal and rostral shell thalamus. (D) The ratio of layer 6 input neurons to all cortical input neurons in MGV and PP/PIL cases. MGV received more cortical input neurons from Au1, AuV, and S1/S2 compared to PP/PIL. (E) The ratio of layer 5 input neurons to all cortical input neurons in the four pathways. No significant difference was detected among the pathways. (F) The ratio of layer 5 input neurons to all cortical input neurons in MGV and PP/PIL cases. Although not statistically significant, PP/PIL tended to receive more layer 5 input from AuD, AuV, and S1/S2. (G) Correlation of the ratio of cortical input neuron layer locations with that of the starter nucleus. Although weak, MGV and Po were positively correlated with layer 6, while PP/PIL and BIC were positively correlated with layer 5. (H) Schematic representation of the results, summarizing the layer-specific distribution of input to different pathways. Statistical parameters related to this Figure are listed in Tables S10–S17.

It has been proposed that in the auditory cortex, layer 6 primarily provides corticothalamic projections to the MGV, while layer 5 and the upper layers project to higher-order thalamic nuclei.^9^^,^^30^ Previous research indicated that the MGD predominantly receives input from layer 6, whereas PP/PIL receives more input from layer 5,^18^ suggesting hierarchical differences among auditory thalamic nuclei. To elucidate the relationship between thalamic regions and cortical layers, we first calculated the ratio of input from each cortical layer relative to all cortical input (Figures 8A and 8B). Among the four pathways, the core pathway tended to receive more input from layer 6 and less from layer 5 compared to the caudal pathway (*p* = 0.058 and 0.075, d = 1.33 and −1.25, respectively; Figure 8A). When focusing on the core and PP/PIL, these trends became statistically significant (*p* = 0.019 and 0.030, d = 2.02 and −1.83, respectively; Figure 8B).

We then calculated the ratio of layer 5 and layer 6 inputs from each cortical area relative to all cortical input neurons (Figures 8C–8F). The core thalamus received a significantly higher ratio of layer 6 input from Au1 compared to the caudal and ventral shell pathways, from AuV compared to the caudal and rostral shell pathways (Figure 8C), and from S1/S2 compared to PP/PIL (Figure 8D), suggesting layer 6 dominant input to the MGV. Thalamic regions of the ventral pathway received more layer 6 input from AuV than other shell pathways (Figure 8C). Although the ratio of layer 5 input was not significantly different, the caudal and PP/PIL pathways tended to receive more input from layer 5 in non-primary auditory areas (AuV and AuD) and S1/S2 (Figures 8E and 8F).

Next, we calculated Spearman’s correlation coefficients between the ratio of input neurons in each cortical layer and that of starter neurons in the starter nuclei (Figure 8G). MGV, MGD, and Po showed positive correlations with layer 6, while PP/PIL and BIC exhibited positive correlations with layer 5. Although MGD and several starter nuclei showed positive correlation with layers 2–4, contribution of upper layers are negligible as the percentage of input from these upper layers is very small compared to deeper layers (percentage of input neurons to all cortical input neurons: layer 2–3, 1.06% ± 2.49; layer 4, 1.77% ± 5.23; layer 5, 26.00% ± 26.78; layer 6, 71.22% ± 29.51; *N* = 29). These findings align with the proposed organization, though the correlations were weak. Taken together, these results summarize the input from each layer to the four pathways (Figure 8H).

### Terminal fields formed by thalamic regions associated with the pathways differed

**Figure 9:**
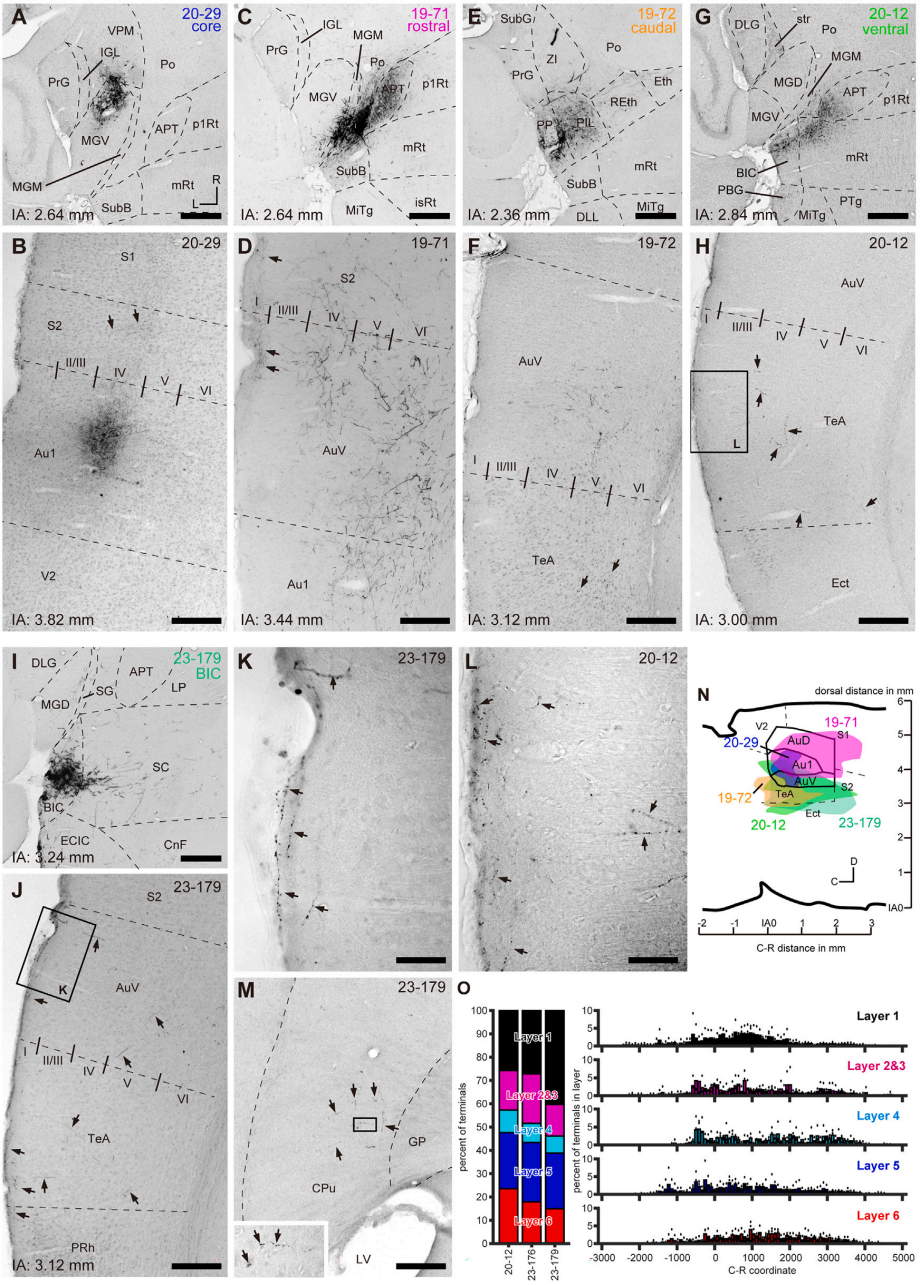
Fiber distribution from auditory thalamic regions to the neocortex (A and B) Core-targeted case (20–29). eGFP-positive somata were restricted to a small region of the MGV (A), and eGFP-positive fibers were confined to layer 4 of a small region in Au1 (B). (C and D) Rostral shell case (19–71). eGFP-positive somata were primarily located in MGM and Po (C), and eGFP-positive fibers were found in all layers of S2, AuV, and Au1 (D). (E and F) Caudal shell case (19–72). eGFP-positive somata were mainly distributed in PP and PIL (E), and eGFP-positive fibers were sparsely distributed in the deeper layers (4–6) of AuV and TeA (F). (G and H) Ventral shell case (20-12). eGFP-positive somata were mostly located in BIC, mRt, and APT (G), and eGFP-positive fibers were sparsely distributed in layer 1 of TeA (H). (I and J) Case (23–179) that received Sindbis pal-eGFP virus injection into the BIC. eGFP-positive somata were restricted to the BIC (I), and eGFP-positive fibers were sparsely distributed in AuV, TeA, and PRh, terminating in layer 1 of these areas (J). (K and L) Higher magnifications of the boxed regions in J and H, respectively. (M) In the same case, terminal plexuses were observed in the tail of the CPu. A higher magnification of the boxed region is shown at the top right. LV, lateral ventricle. Arrows indicate varicose fibers. Scale bars: 400 μm (A, C, E, G, and I), 200 μm (B, D, F, H, J, and M), and 50 μm (K and L). (N) Lateral view of 3D-reconstructed terminal fields of core (20–29, blue), rostral (19–71, magenta), caudal (19–72, orange), ventral (20–12, green), and BIC injection (23–179, pale green) cases merged with sagittal sections and outlines of auditory areas (AuD, Au1, and AuV) as described in the mouse atlas (Paxinos and Franklin, 2019). Terminal fields of all cases overlapped to some extent with the auditory areas. Fibers of the core case are confined to Au1 and AuV, while fibers of rostral, caudal, and ventral cases spread to neocortical areas rostral, caudal, and ventral to the auditory regions, respectively. (O) Distribution of axonal terminals in each cortical layer of three cases that received injections into the BIC. Bar graphs represent the percentage of terminals in layer 1 (black), 2 and 3 (magenta), 4 (cyan), 5 (blue), and 6 (red). Histograms illustrate the caudal-to-rostral distribution of terminals in each layer, showing the widespread distribution of BIC terminals. Dots represent individual case ratios, and bars represent the average ratio.

Although the TRIO experiment provides insight into the origins of thalamocortical projections to a given cortical field, it does not reveal the arborization patterns of thalamocortical axons. To examine the terminal distribution of thalamocortical fibers from each pathway, we analyzed the distribution of eGFP-positive thalamic axons in the neocortex. We confirmed that eGFP-positive fibers were present in auditory cortical areas (Au1, AuV, and AuD) in fluorescent sections across all cases. We then conducted bright-field immunohistochemistry for eGFP to further investigate the distribution of thalamocortical axons. For the analysis, we selected 11 cases where the distribution of eGFP-positive neurons was restricted (Figures 9A, 9C, 9E, and 9G) and similar to the distribution of starter neurons.

As expected, the distribution of eGFP-positive fibers corresponded to the injection site of rgAAV (Figure 9N). In core cases, eGFP-positive fibers were confined to Au1 and AuV. In a case where the injection was restricted, fibers terminated in a small region of layer 4 in Au1 (Figure 9B) and AuV.

In rostral shell cases, which contained more starter neurons in the Po, eGFP-positive fibers were distributed in AuD, Au1, AuV, and extended into cortical areas rostral to the auditory cortex, including the primary and secondary somatosensory cortices (S1 and S2) (Figure 9N). At higher magnification, fibers were distributed across all layers of AuV and S2 (Figure 9D) as well as AuD and Au1. These findings are consistent with previous studies showing the diffuse distribution of Po axons across multiple cortical fields, including motor, somatosensory, auditory, insular, and ectorhinal areas.^31^

In caudal shell cases, which contained more starter neurons in PP/PIL, eGFP-positive fibers were distributed in AuV and extended into cortical areas caudal and ventral to AuV, such as the temporal association (TeA), ectorhinal (Ect), and perirhinal (PRh) cortices. At higher magnification, fibers were sparsely distributed in the deeper layers (4–6) of AuV and TeA (Figure 9F). These observations are consistent with previous studies indicating that PP/PIL axons have a diffuse distribution across various cortical areas, terminating more caudally and being more related to caudal TeA than SG and MGM axons.^12^^,^^13^

In ventral shell cases, where eGFP-positive cells were primarily located caudal (BIC and subB) to the MG (Figure 9G), eGFP-labeled fibers were sparsely distributed and terminated mainly in layer 1 of a broad auditory cortical area, including Au1, AuV, and the area ventral to AuV, namely TeA (Figure 9H, L), Ect, and PRh.

Although starter neurons were found in the BIC in ventral injection cases (Figures 2B and 2F), it has not been previously reported that the BIC gives rise to cortical projection fibers. To confirm whether the BIC indeed projects to the neocortex, we injected recombinant Sindbis virus, which is a well-established anterograde tracer,^24^^,^^26^^,^^31^^,^^32^^,^^33^^,^^34^^,^^35^^,^^36^ into the BIC. In a case where the injection was restricted to the BIC (23–179; Figure 9I), labeled fibers were found in Au1, AuV, and extended ventrally, overlapping with the terminal distribution of the ventral shell cases (Figure 9N). Detailed examination revealed that labeled fibers ascended from the white matter to layer 1 of AuV, TeA, and PRh (Figure 9J), as well as Au1. BIC axons formed terminal puncta in layer 1 (Figure 9K) and other layers. Outside the neocortex, axonal terminals were found in the tail of the CPu (Figure 9M). We plotted varicosities in neocortical areas and analyzed their distribution across layers and rostro-caudal regions in three cases of rabies and Sindbis experiments. Axonal terminals were found in all layers (Figure 9O, left), with widespread distribution along the rostro-caudal axis (Figures 9P and 9O, right). Statistical analysis revealed that the number of terminals in layers 1 and 5 was significantly higher, and in layers 2–3 and 4 significantly lower, than expected based on the proportional area of each layer (*p* < 0.001, population proportion test). These results demonstrate that each shell pathway terminates differently in the neocortex (Figure 9N), and that the BIC directly projects to the neocortex, preferentially terminating in layer 1 of the auditory cortex and ventral cortical areas.

## Discussion

We employed the TRIO method to investigate the input/output relationships of thalamic nuclei associated with auditory pathways. Our findings revealed that the MGV receives inputs not only from auditory sources but also from other modalities, and sends projections to core auditory cortical fields. Shell cortical fields were categorized into rostral, ventral, and caudal regions based on their relative locations to the core field. By comparing input ratios across pathways and conducting correlation analyses between input nuclei and between input and starter nuclei, we identified significant differences in the composition of input sources among auditory thalamic regions. Furthermore, the composition of inhibitory tectothalamic cell types varied across pathways, LG neurons being the dominant cell type in the core pathway. Thalamocortical axons from each pathway terminated in distinct cortical regions and layers. Taken together, we conclude that the auditory system is composed of parallel pathways that are segregated at least partially from the input to the thalamus.

### Comparison with previous anatomical studies

Cai and colleagues^18^ explored the input/output relationships of the non-lemniscal auditory thalamus using Cre mouse lines and Cre-dependent monosynaptic tracing. They classified animals into two groups: MGD/SG and PP/PIL. Their findings showed that PP/PIL received more input from non-auditory structures, such as the basal nuclei, hypothalamus, and ZI, compared to MGD/SG. Although they did not examine the MGV, our correlation matrix (Figure 7) supports their observations regarding inputs to PP/PIL. Traditionally, it has been believed that the MGV receives input primarily from the ICC, while shell auditory thalamic nuclei receive input from the IC cortex.^37^^,^^38^ However, our dataset showed that MGV-main cases also received substantial input from the IC cortex (Figures 5A and 5B). A recent study by Liu and colleagues^39^ examined the projections of PV- and somatostatin (SOM)-positive cells in the mouse IC. They found that both cell types are distributed across all IC subdivisions, with PV neurons being abundant in the ICC and SOM neurons in the IC cortex. Regardless of their location, PV and SOM neurons predominantly project to the primary (MGV) and secondary auditory thalamic nuclei, respectively. These findings, along with ours, suggest that core/shell distinctions may not be solely based on subdivisions but are likely more related to specific cell types involved in the pathways.

### BIC as a thalamic nucleus

One of the unexpected discoveries in this study was that the BIC sends ascending projection fibers to the neocortex, as confirmed by both anterograde (Figure 9) and retrograde (Figures 2B_1_ and 2F) tracing experiments. Given its location adjacent to the MGV, its diffuse projections to layer 1 of both the association and auditory cortices, and its cortical afferent mainly from layer 5, we propose that the BIC should be considered a higher order shell auditory thalamic nucleus. This is consistent with the fact that the BIC receives ascending inputs from LG neurons,^27^ which contribute to tectothalamic projections.^22^^,^^25^^,^^28^

According to the classification criteria proposed by Clasca,^40^ thalamocortical projections can be divided into six classes based on their targets and axonal distribution patterns: unifocal thalamocortical, multifocal thalamocortical, subpial thalamocortical, anterior thalamostriatal, midline thalamostriatal, and posterior thalamostriatal. In the auditory system, MGV, MGD, Po, and PIL are classified as unifocal thalamocortical, multifocal thalamocortical, subpial thalamocortical, and posterior thalamostriatal, respectively. Based on these criteria, it is likely that BIC projections should be classified as posterior thalamostriatal, as they diffusely project to multiple cortical areas and layers, and also target basal nuclei.

### Bypass projection from lower auditory brainstem nuclei to shell thalamus

In this study, we identified direct projections from lower auditory brainstem nuclei, specifically the cochlear nuclei, SOC, and NLL, to the thalamus. Notably, ILL/VLL/PL projected more densely to the ventral shell pathway compared to the core and caudal pathways. The correlation matrix (Figure 7) also indicated a relationship between DLL and the caudal pathway, suggesting that bypass projections may play a more significant role in shell pathways than in the core pathway.

Previous studies have demonstrated direct projections from the dorsal cochlear nucleus (DCN)^41^ and DLL^42^ to the MGM. These projections are non-topographic, and it has been suggested that they are not involved in sound frequency discrimination but are instead responsible for conveying multimodal information with short latency. We speculate that SOC projections to the shell pathway may function similarly to DCN and DLL projections.

### Cytoarchitectural auditory cortex vs. functional imaging auditory field

**Figure 10:**
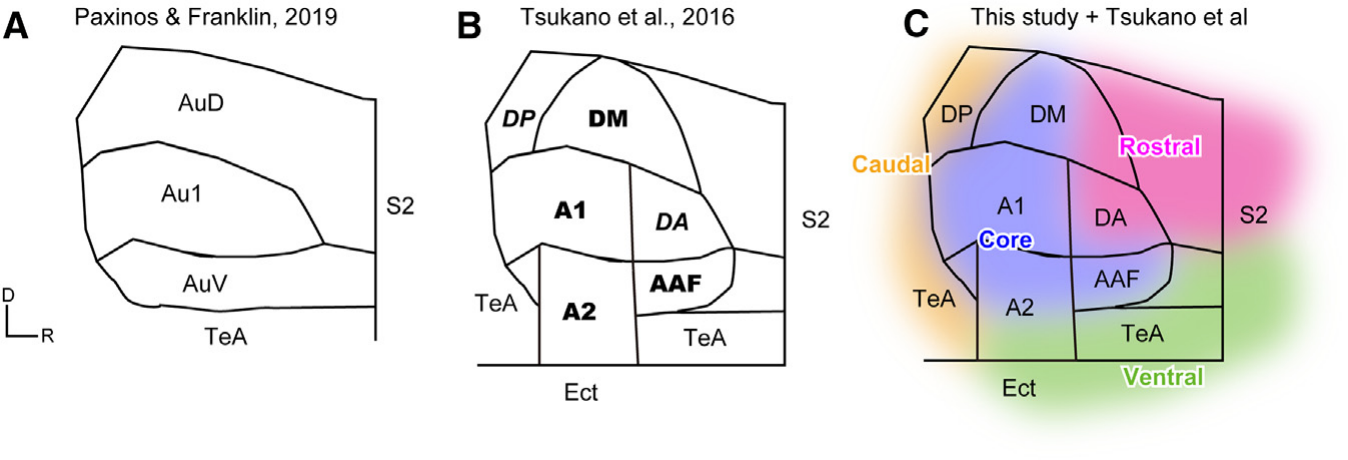
Delineation of auditory-related areas in previous and current studies (A) Cytoarchitectonic delineation described in Paxinos and Franklin (2019). Three auditory areas—AuD, Au1, and AuV—are shown, located posterior to S2 and anterior and dorsal to TeA. (B) Summary of functional imaging studies by Tsukano and colleagues. The delineations by Tsukano et al. (2016) were merged onto the Paxinos and Franklin atlas. Areas with bold labels (DM, A1, AAF, and A2) exhibited tonotopicity, while those with italic labels (DA and DP) responded to frequency-modulated sweeps. (C) The rostral, ventral, and caudal shell areas identified in the current study were superimposed on (B). As the exact borders of these regions could not be determined in the current study, the correspondence between the areas identified by Tsukano et al.^5^ and this study is speculative.

In this study, we utilized the standard atlas by Paxinos and Franklin^43^ (Figure 10A). In contrast, Tsukano and colleagues^5^ correlated cytoarchitectural delineations with functional maps of the mouse auditory cortex (Figure 10B). The key differences include the subdivision of the caudal part of AuD into tonotopic DM and non-tonotopic dorsoposterior (DP) fields, the subdivision of Au1 into non-tonotopic dorsoanterior (DA) and tonotopic A1 proper fields, and the subdivision of the caudal AuV into the tonotopic AAF and secondary auditory field (A2), with a ventral extension of A2.

Based on our results and previous studies, it is likely that the cortical fields associated with the core pathway correspond to DM, A1, A2, and AAF. The cortical fields related to the rostral shell pathway likely include parts of S2 and DA, while those associated with the ventral shell pathway likely involve S2, IAF, anterior TeA, and Ect. The caudal shell pathway likely corresponds to DP and caudal TeA (Figure 10C). The outer limits of the shell cortical field could not be precisely defined in this study.

### Cell types of IC GABAergic projection neurons

There is ongoing debate regarding the cell types of GABAergic tectothalamic projection neurons in the IC. Ito and colleagues^22^ injected a retrograde tracer into the rat MGV and found that most tracer-labeled GABAergic neurons in the ICC were LG neurons. In contrast, a study in guinea pigs^44^ indicated that SmG neurons are the dominant type of GABAergic tectothalamic projection neurons. The discrepancy may stem from species differences and/or technical factors, such as the use of stereology^45^ in the former study to avoid counting bias. Additionally, the injection sites of the tracer in the MGB could be another factor: in this study, core cases showed a higher percentage of retrogradely labeled LG cells in the ICC, while ventral shell cases showed a lower percentage, with SmG cells accounting for a comparable ratio to LG cells (Figure 6G). The higher ratio of SmG neurons in the tectothalamic projection observed by Beebe and colleagues may result from the inclusion of both shell and core cases in their analysis. It is plausible that GABAergic cell types play different roles in core and shell tectothalamic pathways.

LG neurons show a preference for frequency-modulated tones compared to SmG and GLU neurons, and a single LG neuron projects axons simultaneously to the contralateral IC, lateral lemniscus, and brachium of the IC, with dense local collaterals.^28^ This suggests that a single LG neuron can influence multiple layers of hierarchical auditory processing (e.g., lower brainstem, IC, and MGB), suppress postsynaptic neurons across these layers in response to preferred stimuli, and generate complex responses to frequency modulation across these layers. Our study suggests that neurons in the core pathway are more influenced by LG neuron activity. The core pathway is likely crucial for analyzing sound features, and A1, which is responsible for detecting syllables characterized by amplitude and frequency modulation,^46^ may rely on indirect input from frequency-modulation-sensitive LG neurons via MGV.

LG neurons in the IC cortex may serve a different function. Geis and Borst^27^ performed whole-cell recordings from presumed LG neurons in the dorsal IC cortex and found that they respond to sound with short latency. In contrast, the latency of ICC LG neurons is similar to other cell types.^28^ These findings imply different temporal characteristics of LG neurons between ICC and IC cortex, although current knowledge about physiological properties of LG neurons in the IC cortex is limited. Morphologically, all LG neurons in the dorsal cortex have axon collaterals terminating in the BIC (Geis and Borst, 2013). This finding aligns with our data, suggesting that LG neurons in the IC cortex contribute more to the ventral shell pathway than SmG neurons (Figure 6H).

### Functional considerations

**Figure 11:**
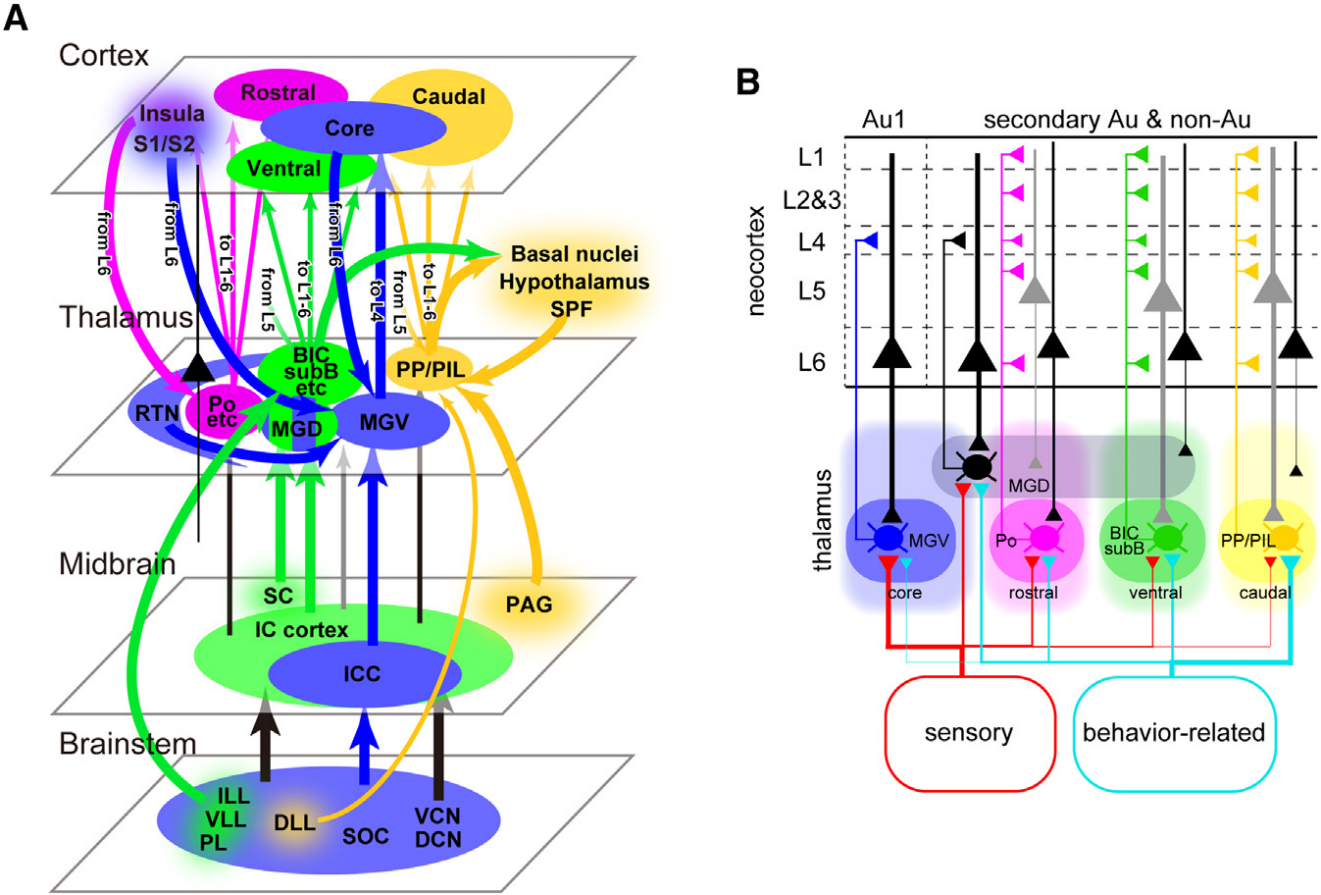
Parallel pathways of auditory circuitry through the brain hierarchy (A) Summary of projections. Colors indicate nuclei and projections associated with each individual pathway. Some nuclei participate in multiple pathways, illustrating the complex and overlapping nature of auditory processing circuits throughout the brain hierarchy. (B) More simplified view based on the functions of nuclei and the hierarchy of thalamo-cortical pathways. Line thickness reflects the strength of the projection. Left part represents thalamic regions that receive more sensory information and feedback from layer 6, and project to layer 4. Right part represents higher-order thalamic regions that receive more behavior-related information and feedback from layer 5, and project to all cortical layers.

We described the core and three shell pathways (Figure 11). Based on their input/output relationships, we can infer the potential functions of each pathway.

Our correlation matrix (Figure 7) clearly demonstrates that the core pathway is involved not only in auditory but also multimodal sensory processing. Multimodal information is provided by descending projections from the somatosensory and insular cortices, as well as the RTN. Sound-responsive neurons in the RTN frequently receive projections from thalamic relay nuclei and neocortical areas of the somatosensory and/or visual systems,^47^ and the firing activities of RTN neurons exhibit complex interactions with auditory, somatosensory, and visual stimuli.^48^^,^^49^ As the RTN is thought to gate sensory information,^50^ the dense projections from sensory cortices and the RTN to the MGV suggest that the core pathway plays a crucial role in selective attention to specific sensory modalities.

The connections of the rostral shell pathway were less defined than those of other shell pathways. It exhibited a weak correlation with input from the insular cortex. The thalamic nucleus strongly related with this pathway is Po. Some Po and MGM neurons associated with the rostral pathway show short-latency responses to sound.^51^ Single Po axons primarily terminate in layers 1, 4, and 5 of motor, somatosensory, auditory, insular, and ectorhinal cortices, with Po neurons terminating more densely in layer 1 than in other layers.^31^ It is likely that Po and the insular cortex, which contain IAF, are reciprocally connected, and their target may include the DM auditory field, which responds strongly to fast frequency-modulated sweeps.^52^ The widespread output of the Po and the short latency of Po neurons suggest that the rostral pathway is suitable for broadcasting the timing of sound.

The ventral shell pathway is characterized by dense input from the IC cortex and SC, both of which are crucial for driving innate escape behaviors in response to loud sounds.^53^ This pathway also receives dense input from the ventral complex of the lateral lemniscus (ILL/VLL/PL). VLL neurons respond accurately to the onset of sound,^54^ with properties inherited from octopus cells that project to the ventral VLL.^55^ The dense input from lateral lemniscal nuclei suggests that the BIC may be driven by short-latency sound input. Projections from LG neurons in the IC cortex to the BIC may serve as another source of short-latency input. Conversely, the BIC, the thalamic nucleus strongly related with this pathway, sends diffuse axons across multiple layers to the ventral TeA and Ect, and has axon collaterals in the tail of striatum. Though the function of the BIC is not fully understood, a recent study showed that it is important for auditory attention during audiovisual tasks.^56^ The TeA is important for maternal recognition of pup calls.^57^ Taken together, the ventral pathway is likely important for learning multimodal tasks, sound-induced behaviors, and the perception of complex sounds.

The caudal shell pathway is distinguished by denser input from the PAG, hypothalamus, caudal basal nuclei, and SPF, than other pathways. The PAG drives innate escape behaviors in response to loud sounds,^53^ while the hypothalamus is critical for various innate behaviors.^58^ Our correlation matrix showed that input from the hypothalamus was positively correlated with input from the amygdala (Figure 7A), highlighting the importance of emotional states in this pathway. The basal nuclei have been implicated in innate escape behaviors triggered by looming sounds.^21^ Regarding the caudal pathway’s output, thalamocortical axons from PP/PIL have been studied using bulk injections of anterograde tracers.^12^^,^^13^ Axons from shell nuclei terminate across layers 1, 4, and 5 of the somatosensory, auditory, insular, and association cortices. Compared to SG and MGM, PP/PIL axons terminate more caudally and are more associated with caudal TeA. Taken together, the caudal pathway is likely to send sound information that is modulated by emotion and behavioral relevance.

One intriguing characteristic of the ventral and caudal shell pathways is that both thalamic nuclei project to the tail of the striatum and receive more layer 5 input from non-primary cortical areas. The tail of the striatum acts as an integrator of auditory and visual information, contributing to the encoding of reinforcement learning.^59^ Layer 5 corticothalamic neurons, known for receiving input from multiple layers and exhibiting nonlinear spiking properties, contrast with layer 6 corticothalamic neurons, which primarily receive input from layer 6 and have regular spiking properties.^60^ This suggests that information conveyed by layer 5 pyramidal neurons is more heavily processed and modified than that from layer 6. These observations imply that the ventral and caudal shell thalamic nuclei are higher-order thalamus acting as channels through which sensory information, refined by higher cortical centers, is relayed for reinforcement learning, particularly in multimodal tasks or emotionally charged events (Figure 11B).

Additionally, the caudal shell pathway stands out by receiving significantly stronger input from the SPF compared to the core pathway. SPF is a dopaminergic nucleus that projects to the auditory midbrain,^17^ thalamus,^18^ and neocortex (Figures 4E and 4L), and is implicated in predictive coding of the auditory environment and the encoding of uncertainty.^61^ Dopaminergic input to the tail of the striatum is thought to signal the prediction or confidence of sound perception.^62^ In rats, the posterior auditory field exhibits the strongest prediction error signals compared to other auditory cortical areas.^63^ Thus, dopaminergic input to the caudal pathway likely introduces a predictive bias to auditory information relayed from the brainstem, enhancing the brain’s ability to process sound in the context of prediction and uncertainty.

### Limitations of the study

We utilized viral tracers to label neurons, and it is known that each virus exhibits tropism, or selectivity in infection.^64^ Although our results largely align with previous anatomical studies, we cannot completely rule out the possibility that the observed labeling patterns were influenced by viral tropism. Another limitation pertains to the correlation analysis we employed to estimate input relationships, a method commonly used in rabies tracing studies.^17^^,^^18^^,^^20^ The advantage of this method is that it allows the inclusion of more cases than “representative” examples, potentially providing a more objective result. However, the downside is that tracer leakage may introduce “noise” into the correlation matrix. A more restricted tracer injection might yield more accurate input relationships.

## Resource availability

### Lead contact

Further information and requests for resources should be directed to and will be fulfilled by the lead contact, Tetsufumi Ito (itot@med.u-toyama.ac.jp).

### Materials availability

This study did not generate new unique reagents.

### Data and code availability

• Data used for quantitative analysis are provided as Data S1 and S2. All the other data reported in this paper will be shared by the lead contact upon request.
• All original codes for quantitative analysis and producing graphs are provided as Data S1 and S2.
• Any additional information required to reanalyze the data reported in this paper is available from the lead contact upon request.

## Acknowledgments

This work was supported by 10.13039/501100001691KAKENHI grants (23H03243, 23H04342, and 20H05950 for T.I., and JP16K11200 for M.O.), the JST FOREST Program (JPMJFR2151 for T.I.), the 10.13039/501100004330Smoking Research Foundation (T.I.), and a grant from 10.13039/100019433Shibuya Science Culture and Sports Foundation (M.O.). We thank Dr. Douglas L. Oliver for critical reading of the manuscript and Dr. Eriko Kuramoto (Kagoshima University) for providing detailed information about thalamocortical system.

## Author contributions

T.I. conceived the study design and wrote the paper. All authors performed experiments, analyzed the data, and edited the manuscript.

## Declaration of interests

The authors declare no competing interests.

## Declaration of generative AI and AI-assisted technologies in the writing process

During the preparation of this work the authors used Chat-GPTo in order to check English grammar. After using this service, the authors reviewed and edited the content as needed and take full responsibility for the content of the published article.

## STAR★Methods

### Key resources table

**Table:** 

REAGENT or RESOURCE	SOURCE	IDENTIFIER
**Antibodies**		

Mouse monoclonal anti-Cre	Merck Millipore	RRID: AB_2085748; MAB3120
Mouse monoclonal anti- glutamic acid decarboxylase 67	Merck Millipore	RRID: AB_2278725; MAB5406
Mouse monoclonal anti- parvalbumin	Sigma-Aldrich	RRID: AB_477329; P3088
Rat monoclonal anti-mCherry	Thermo-Fisher Scientific	RRID: AB_2536611; clone 16D7
Rabbit polyclonal anti-eGFP	Takeshi Kaneko	RRID: AB_2921270
Guinea-pig polyclonal anti- vesicular glutamate transporter 2	Frontier Institute	RRID: AB_2571620; MSFR106280
Guinea-pig polyclonal anti- monomeric red fluorescent protein	Takeshi Kaneko	RRID: AB_2336889
Goat polyclonal anti-calretinin	Merck Millipore	RRID: AB_90764; AB1550

**Bacterial and virus strains**		

retrograde AAV-Ef1α-Cre	Salk Institute Vector Core	RRID:Addgene_55636
AAV-hSyn-FLEX-TVA-P2A-eGFP-2A-G	Salk Institute Vector Core	RRID:Addgene_85225
EnvA-coated ΔG-mCherry rabies virus	Salk Institute Vector Core	N/A
Sindbis-pal-eGFP	Takeshi Kaneko	N/A
Sindbis-pal-mRFP	Takeshi Kaneko	N/A

**Chemicals, peptides, and recombinant proteins**		

dental cement	GC Corporation	GC Fuji Ionomer Type II
silicone elastomer	World Precision Instruments	Kwik-Sil

**Deposited data**		

data for producing Figures 3, 4, 5, 6, 7, and 8	authors	Data S1
data for producing Figure 9	authors	Data S2

**Experimental models: Organisms/strains**		

Mouse: CBA/J	Jackson Laboratory Japan	CBA/J

**Software and algorithms**		

MATLAB	MathWorks	RRID: SCR_001622
Photoshop CS3	Adobe Systems	RRID:SCR_014199
Neurolucida	MBF bioscience	N/A
MATLAB code for producing Figures 3, 4, 5, 6, 7, and 8 (‘analysis.m’)	authors	Data S1
MATLAB code for producing Figure 9 (‘analysis_Fig9P.m’)	authors	Data S2

**Other**		

magnetic speaker	Tucker-Davis Technologies	MF1
1/4-inch Electret condenser microphone	Rion	UC-54
Sound level meter	Rion	NA-40
real-time signal processor	Tucker-Davis Technologies	RP2.1
tiltable head-fix apparatus	Narishige	SG-4N
fluorescent stereo microscope	Olympus	SZX-12
animal body temperature controller	Bio Research Center	BWT-100A
cooled CCD camera	Bitran	BU-60
freezing slide microtome	Yamato Koki	REM-710
stereotaxic apparatus	Narishige	SR-5M-HT
structured illumination fluorescent microscope	Carl Zeiss	Axioimager.M2 plus Apotome.2
upright microscope	Olympus	AX-80
digital camera	Panasonic	DMC-GH-3

### Experimental model and study participant details

#### Experimental design

To label the auditory-pathway-specific neuronal populations, we employed the “tracing the relationship between input and output” (TRIO) method^20^ to functionally identified auditory cortical areas (Figure 1; Figure S1). First, we performed flavin imaging^19^ to identify auditory cortical areas in male mice. After identification, a retrograde serotype of adeno-associated virus (rgAAV-Ef1α-Cre; Salk Institute Vector Core) was injected into a cortical area responding to sound stimuli. The auditory thalamus was then injected with a large amount of a helper virus that promoted the expression of avian leukosis and sarcoma virus subgroup A receptor (TVA), enhanced green fluorescent protein (eGFP), and rabies glycoprotein (G) in the presence of Cre recombinase (AAV-hSyn-FLEX-TVA-P2A-eGFP-2A-G; Salk Institute Vector Core). Both AAV serotypes 8 and 9 were used, with no apparent differences observed, so the data from these two serotypes were combined. Fourteen days later, we injected a G-deleted rabies virus that was coated with an envelope protein of avian leukosis and sarcoma virus (EnvA) and conveys the mCherry gene (EnvA-ΔG-rabies-mCherry; Salk Institute Vector Core).

Through this method, thalamic neurons projecting to an auditory cortical area, confirmed by flavin imaging, were retrogradely labeled with Cre, expressing TVA, eGFP, and G. The rabies virus used infected only the TVA-expressing cells, which also expressed eGFP, causing the cells to express mCherry and produce rabies viral particles containing G. Neurons expressing both eGFP and mCherry were termed “starter neurons” (yellow cells in Figure S1). The rabies virus produced by starter neurons was transsynaptically transmitted to neurons synapsing onto the starter neurons, promoting the expression of mCherry. These transsynaptically labeled neurons, expressing only mCherry and not eGFP, were designated as “input neurons” (red cells in Figure S1). Nuclei containing starter or input neurons were referred to as "starter nuclei" or "input nuclei," respectively. In this study, we analyzed the relationship between the injection sites of rgAAV-Cre and the distribution of starter and input neurons.

#### Animals

Seventy adult male CBA/J mice (body weight: 13-32 g) were used for this study. We exclusively used male mice, as the estrus cycle has been shown to affect auditory-evoked cortical activity.^65^^,^^66^ Of these, sixty-two were used for TRIO experiments, three for control experiments (omitting the injection of rgAAV-Ef1α-Cre), and five for anterograde tracing experiments. Mice were housed in a temperature- and humidity-controlled room under a 12h/12h light/dark cycle, with free access to food pellets and water. After the experiments, mice were euthanized with a lethal dose of pentobarbital sodium (150 mg/kg, i.p.) and perfused for histological analysis (detailed below). All experiments were conducted in accordance with the institutional guidelines at Kanazawa Medical University (Approval no. 2018-10), University of Toyama (G2023MED-14 and A2023MED-01), and the Guidelines for Proper Conduct of Animal Experiments by the Science Council of Japan. Every effort was made to minimize animal use and suffering.

### Method details

#### Sound production

Sounds were delivered through a closed system using a magnetic speaker (MF1, Tucker-Davis Technologies, Alachua, FL, USA) and hollow ear bars. The sound delivery system was calibrated for frequencies between 700 Hz and 70,000 Hz. For calibration, the ear bar was connected to a 1/4-inch electret condenser microphone (UC-54, Rion, Tokyo, Japan) via a silicone tube. Pure tones (700 Hz to 70,000 Hz in 1/3 octave steps) and band noise (700 to 55,603 Hz) were generated using a real-time signal processor (RP2.1, Tucker-Davis) and played through the speakers. Sound levels were measured with a level meter (NA-40, Rion) connected to the microphone. Signals from the level meter were recorded at 200 kHz, and harmonic distortion was measured using fast-Fourier transformation. Only sounds with harmonic distortion below 40 dB were used, ensuring linearity in the sound delivery system without distortion.

#### Surgery, flavin imaging, and viral injection

Mice were anesthetized using a mixture of medetomidine (0.3 mg/kg), midazolam (4.0 mg/kg), and butorphanol (5.0 mg/kg) and fixed in a tiltable head-fix apparatus (SG-4N; Narishige, Tokyo, Japan). After removing the skin over the cranium, a screw mounting base was attached using dental cement (GC Fuji Ionomer Type II, GC Corporation, Tokyo, Japan). Once the cement hardened, the head was fixed to a brass bar attached to the tiltable head-fix apparatus. The ear bar of right side was replaced with a hollow ear bar connected to a speaker. Liquid paraffin was applied to the left parietal bone to make it transparent for functional imaging, and the head was positioned so that the parietal bone and temporal lobe were parallel to the horizontal plane. Flavin imaging was performed under a fluorescent stereo microscope (SZX-12, Olympus, Tokyo, Japan) using sound stimuli.

Flavin imaging followed the method of Tsukano et al.^67^ The temporal cortex was illuminated with a Hg lamp filtered through a GFP filter during sound presentation. As flavoprotein autofluorescence reflects neuronal activity via activity-dependent oxygen metabolism,^19^ neuronal activity was reconstructed from images taken during sound presentation. The animal’s body temperature was maintained at 37°C using an animal body temperature controller (BWT-100A; Bio Research Center, Nagoya, Japan).

We used 5 kHz, 15 kHz, and 40 kHz tone bursts modulated at 20 Hz. Each sound stimulus lasted 500 ms with a 25-second interstimulus interval. Sound pressure levels ranged from 50 to 70 dB SPL. Amplitude-modulated broadband noise was used to activate broader cortical areas. A 5-ms rise and fall with a linear slope was applied to attenuate click noise, and each stimulus was repeated 20 times. Temporal cortex images were taken at 10 frames per second with a cooled CCD camera (BU-60, Bitran, Saitama, Japan). Image acquisition started 500 ms (5 frames) before sound onset. Readouts from the CCD were binned at 5×5, resulting in 128×168 pixel images with a 16-bit dynamic range. The first 5 frames were averaged as the baseline, and the baseline was subtracted from each image, then divided by the baseline to measure the fluorescence change (Figures 1A–1C). Significant activity was defined as the mean signal minus two standard error of the mean (s.e.m.) above zero (Figures 1D and 1E).

After imaging, the area of significant activation at 0.9 seconds (400 ms after sound onset) for the 5 kHz, 15 kHz, and 40 kHz tone bursts was identified as the auditory cortical area. Prior research has shown that AAF and A1 activities and tonotopic organization can typically be detected 0.4-0.5 s after stimulus onset.^68^

A small hole was drilled in the skull over the activated area (Figures 1E and 1F). A glass micropipette filled with rgAAV-Ef1α-Cre was inserted into the center of the hole at a depth of 500 μm, and 76-300 nl of virus was injected using a nanoliter injector (Nanoject II, Drummond Scientific Company, Broomall, PA, USA). The mouse was then fixed in a stereotaxic apparatus (SR-5M, Narishige), a hole was drilled in the parietal bone, and a glass micropipette filled with AAV-hSyn-FLEX-TVA-P2A-eGFP-2A-G was inserted at coordinates 1.9 mm lateral, 0.7 mm rostral, and 2.5 mm dorsal from the interaural zero point. A 500 nl virus solution was injected using a custom-made injector driven by N_2_ gas pressure. The brain surface was covered with silicone elastomer (Kwik-Sil; World Precision Instruments, Sarasota, FL, USA), atipamezole (0.375 mg/kg) was injected subcutaneously, and the animal was returned to its home cage.

Fourteen days after AAV injection, the animals were anesthetized again, and 500 nl of EnvA-deltaG-rabies-mCherry was injected into the site of the previous helper virus injection. The procedure was the same as described above. Seven days after rabies virus injection, animals were euthanized, perfused with 4% paraformaldehyde in 0.1M phosphate buffer (PB, pH 7.4), and the brains were processed for more than one day at 4°C. The brains were embedded in a gelatine block composed of 20% gelatine, 30% sucrose, and 0.1M PB (pH 7.4). After the gelatine hardened, the blocks were fixed with a fixative composed of 4% paraformaldehyde, 30% sucrose, and 0.1M PB (pH 7.4). Serial horizontal sections at a thickness of 40 μm were cut with a freezing slide microtome (REM-710, Yamato Koki, Saitama, Japan). Every six section was collected in a vial filled with 2% sodium azide in 0.05M phosphate-buffered saline (PBS, pH 7.4) and stored at 4°C.

After perfusion, the exact location of the rgAAV-Cre injection was identified. This was determined by combining activity patterns from flavin imaging, anatomical landmarks such as blood vessels on the cortical surface and bony sutures, and images taken after the craniotomy (Figures 1E and 1F). These images were further superimposed with those from other animals, using both activity patterns and anatomical landmarks. Squamous and lambdoid sutures were particularly reliable for image superimposition across animals, while surface veins showed more variability and were used for fine adjustments. Two consistent veins were used as landmarks: the dorsal middle branch of the caudal rhinal vein, which ran rostroventrally across the parietal cortex, and the dorsocaudal branch of the caudal rhinal vein, which ran caudoventrally across the occipital cortex (Xiong et al., 2017). Based on these images, injection sites were classified into core, rostral, ventral, and caudal shell fields (Figures 1H and 1I). Despite the publication of an atlas correlating flavin imaging auditory fields with anatomically defined auditory cortical areas,^5^ discrepancies with commonly used brain atlases were noted, particularly regarding the boundary of the primary auditory field. Therefore, in this study, the term “A1” refers to regions identified through flavin imaging, while “Au1” refers to anatomically defined regions.

#### Anterograde tracing experiment using Sindbis virus

To investigate the axonal arborization of neurons in the nucleus of the brachium of the inferior colliculus (BIC), we injected recombinant Sindbis virus, which carries a membrane-targeted fluorescent protein gene,^32^ into five mice. The mice were anesthetized with a mixture of medetomidine (0.3 mg/kg), midazolam (4.0 mg/kg), and butorphanol (5.0 mg/kg) and fixed in a stereotaxic apparatus (SR-5M-HT, Narishige). A small hole was drilled into the skull, and a glass micropipette filled with viral solution was inserted. Between 100 to 500 nanoliters of Sindbis pal-eGFP and pal-mRFP viruses were injected into the BIC on both sides, with Sindbis pal-eGFP into one side and Sindbis pal-mRFP into the other. After a subcutaneous injection of atipamezol, the animals were returned to their home cages.

After 48 hours, the animals were euthanized with a lethal dose of pentobarbital sodium (150 mg/kg, i.p.), perfused with 4% paraformaldehyde in 0.1M PB, and their brains were sectioned as described above.

#### Antibodies

We used mouse monoclonal antibodies for Cre recombinase (RRID: AB_2085748; MAB3120, Merck Millipore, Burlington, MA, USA), glutamic acid decarboxylase 67 (GAD67; RRID: AB_2278725; MAB5406, Merck Millipore), and parvalbumin (PV; RRID: AB_477329; P3088, Sigma-Aldrich). We also used rat monoclonal antibodies for mCherry (RRID: AB_2536611; clone 16D7; Thermo-Fisher Scientific, Waltham, MA, USA), rabbit polyclonal antibodies for eGFP (gift from Dr. Takeshi Kaneko; RRID: AB_2921270),^69^ and guinea pig polyclonal antibodies for vesicular glutamate transporter 2 (VGLUT2; RRID: AB_2571620; MSFR106280, Frontier Institute, Ishikari, Japan) and monomeric red fluorescent protein (mRFP; gift from Dr. Takeshi Kaneko; RRID: AB_2336889).^70^ Additionally, a goat polyclonal antibody for calretinin (CR; RRID: AB_90764; AB1550, Merck Millipore) was employed.

The immunogen for the anti-mRFP antibody was a recombinant mRFP, a modified form of the DsRed fluorescent protein from *Discosoma* coral.^71^ This antibody cross-reacts with other modified forms of DsRed, such as mCherry. The anti-eGFP antibody’s immunogen was a recombinant eGFP, and the anti-GAD67 antibody’s immunogen was recombinant GAD67. Specificity for Cre, mCherry, mRFP, and eGFP antibodies was confirmed by the absence of labeling in wild-type mouse brain sections. The specificity of the anti-GAD67 antibody was verified in a prior study,^33^ while the anti-VGLUT2 antibody’s specificity was confirmed by a single band at 60 kDa in a Western blot (described in the manufacturer’s sheet). The anti-CR antibody used a rat calretinin as the immunogen, and the anti-PV antibody used a frog muscle parvalbumin; specificity was determined by ELISA. Additionally, labeling patterns for PV and CR antibodies were consistent with previous studies.^72^^,^^73^

#### Histology

To examine the distribution of starter and input neurons, we performed fluorescent immunohistochemistry for mRFP and Cre. Given that the leaky expression of the floxed gene poses challenges in monosynaptic tracing, it is crucial to exclude neurons without Cre expression from being considered starter neurons. Typically, neurons with leaky floxed gene expression do not show detectable eGFP fluorescence under microscopy, and rabies glycoprotein expression is insufficient for transsynaptic transport. However, leaky TVA expression can still allow infection by EnvA-coated rabies.^74^ As such, we avoided eGFP immunohistochemistry to prevent misidentifying leaky neurons as starter neurons, relying instead on native eGFP fluorescence imaging.

Every sixth serial section was incubated overnight with mouse anti-Cre (1:1000) and guinea pig anti-mRFP (0.5 μg/ml), diluted in incubation buffer composed of 1% normal donkey serum, 0.3% Triton X-100, 0.2% sodium azide, and 0.05M phosphate-buffered saline (PBS), at room temperature. Sections were washed three times with PBS, incubated for 1 hour with Cy5-conjugated donkey anti-mouse IgG (1:200; Jackson ImmunoResearch, West Grove, PA, USA), RhodamineRed-conjugated donkey anti-guinea pig IgG (1:200; Jackson ImmunoResearch), and Neurotrace435/455 (1:400; Thermo-Fisher). After three washes with PBS, sections were mounted on coated glass slides, air-dried, and coverslipped with 1,4-diazabicyclo[2.2.2]octane (DABCO).

To identify cell types of input neurons in the IC, we performed immunolabeling with VGLUT2 and GAD67.^22^ Large GABAergic (LG) neurons were identified by GAD67-positive cell bodies encircled by VGLUT2-positive terminals, while small GABAergic (SmG) neurons lacked VGLUT2-positive axosomatic rings. Putative glutamatergic neurons were identified by the absence of GAD67 labeling in somata. Every sixth serial section was incubated overnight with mouse anti-GAD67 (1:1000), rat anti-mCherry (1:500), and guinea pig anti-VGLUT2 (1:1000) diluted in incubation buffer at room temperature. The sections were washed three times with PBS and incubated for 1 hour with Cy5-conjugated donkey anti-mouse IgG (1:200; Jackson ImmunoResearch), RhodamineRed-conjugated donkey anti-rat IgG (1:200), and AlexaFluor488-conjugated donkey anti-guinea pig IgG (1:200). Sections were mounted on glass slides, air-dried, and coverslipped with DABCO.

To study the distribution of eGFP-positive fibers in the neocortex, we performed bright-field immunohistochemistry for eGFP. Bright-field immunohistochemistry was also performed to delineate core and shell brain regions using antibodies for parvalbumin and calretinin, respectively. Every sixth serial section was incubated overnight with either rabbit anti-eGFP (0.2 μg/ml), mouse anti-PV (1:1000), or goat anti-CR (1:1000). Sections were washed three times with PBS, incubated for 1 hour with biotinylated donkey antibodies for rabbit IgG, mouse IgG, or goat IgG (1:200; Jackson ImmunoResearch), followed by three PBS washes, and incubation with avidin-biotinylated horseradish peroxidase complex. Bound peroxidase was visualized using a nickel diaminobenzidine reaction. Sections were mounted on glass slides, air-dried, dehydrated, cleared with Histo-Clear (National Diagnostics, Atlanta, GA, USA), and coverslipped with Entellan (Merck Millipore).

#### Image acquisition

Fluorescent sections were imaged using a structured illumination fluorescent microscope (Axioimager.M2 plus Apotome.2, Carl Zeiss Microscopy, Jena, Germany) equipped with four light-emitting diodes (LED; Colibri 5, Zeiss). Neurotrace435/455 was excited by a 385-nm LED, with emitted fluorescence filtered using a 410-440-nm band-pass filter. AlexaFluor488 and eGFP were excited by a 475-nm LED, with emitted fluorescence filtered using a 499-529-nm band-pass filter. RhodamineRed and mCherry were excited by a 555-nm LED, with emitted fluorescence filtered using a 580-605-nm band-pass filter. Cy5 was excited by a 630-nm LED, with emitted fluorescence filtered using a 659-nm low-pass filter.

To map the distribution of starter and input neurons, every sixth section was fully imaged. Tiled images of brain sections were acquired using a 10× lens (NA = 0.45) at a resolution of 1.82 μm/pixel. For IC cell type identification, tiled IC images were acquired using a 63× lens (NA = 1.4) at a resolution of 0.36 μm/pixel. The montage of tiled images was created using the "Stitching" method in the microscope control software (Zen, Carl Zeiss).

Chromophorically stained sections were imaged using an upright microscope (AX-80, Olympus, Tokyo, Japan) with a digital camera (DMC-GH-3, Panasonic, Osaka, Japan). Minimal adjustments were made using Photoshop CS3 software (Adobe Systems, San Jose, CA; RRID:SCR_014199).

### Quantification and statistical analysis

#### Data analysis

For the quantitative analysis of microscopic images, montage images of every sixth serial section were imported into Neurolucida software (MBF Bioscience, Williston, VT, USA). The section outlines and the borders of each brain region were manually drawn, and labeled cells were plotted. Brain regions were identified by comparing Nissl cytoarchitecture with a brain atlas.^43^ Additionally, "core" and "shell" thalamic and cortical areas were identified using PV- and CR-immunostained sections, respectively.^72^^,^^73^ The analyzed data were exported to a custom MATLAB R2021b script with the Statistics and Machine Learning Toolbox (version 12.2; Mathworks, Natick, MA, USA) for further analysis.

To quantify the distribution of starter and input neurons, eGFP- and mCherry-positive somata were manually plotted in Neurolucida software. Neurons double-positive for both eGFP and mCherry were identified as starter neurons, while mCherry-only-positive neurons were classified as input neurons. Cre-immunoreactivity was not used to identify starter neurons due to the high sensitivity of Cre recombinase, as even undetectable amounts can suffice for recombination at floxed sites. In some cases, we did not see the overlap of Cre on eGFP/mCherry double-positive neurons, but mCherry-positive neurons were found far from the injection site. As the aim of the study is to clarify the source of the brain-wide inputs to thalamic regions, we excluded animals with starter neurons in the cerebral cortex or basal forebrain from the analysis.

As noted in the ***Histology*** section, mCherry-positive neurons located near the helper virus injection site could be erroneously identified as input neurons due to leaked TVA expression. Therefore, nuclei consistently adjacent to the pipette track of the helper and rabies viruses (BIC, subbrachial nucleus [subB], ethmoid nucleus [Eth], retroethmoid nucleus [REth], hippocampus [HIP], intergeniculate leaflet [IGL], pregeniculate nucleus [PrG], MGV, MGD, MGM, SG, PP, and PIL of the ipsilateral side) were excluded from the input neuron count (see Table S1). Additionally, mCherry-positive neurons within the same nuclei as starter neurons were excluded from the analysis. In cases where starter neurons were present in a nucleus, input-cell counts from that nucleus were marked as data-deficient ("NaN").

A control experiment was performed on three mice without rgAAV-Cre injection to assess the efficiency of leaked TVA expression in facilitating rabies infection. The number of mCherry-positive neurons in the thalamus was 26, 60, and 181, while the number outside the thalamus was 0, 0, and 14, respectively. Based on these findings, we considered cases with fewer than 200 input neurons expressing mCherry alone to be unreliable and excluded them from further analysis. Following this exclusion, 29 cases remained for subsequent analyses.

The ratio of starter neurons in each nucleus was calculated for each case by dividing the number of starter neurons in a given nucleus by the total number of starter neurons. Similarly, the ratio of input neurons in each case was determined. For ratio calculations, neighboring nuclei with similar functions or cytoarchitecture were grouped together (Table S1). Nuclei near the midline with similar input neuron counts across sides (e.g., SC, PAG, and hypothalamus) were also grouped together. Input nuclei with a mean input ratio below 0.5% and a maximum input ratio below 1% were excluded from further analysis. All nuclei containing mCherry-positive neurons are listed in Table S1.

For analyzing IC neuron cell types labeled with mCherry, montage images of every sixth serial section were imported into Neurolucida software. mCherry-positive somata were manually plotted, and the somata were classified into LG, SmG, and non-GABAergic, putative glutamatergic (GLU) neurons based on established criteria.^22^

#### Statistical analysis

All statistical analyses were conducted using MATLAB R2021b with the Statistics and Machine Learning Toolbox (version 12.2; Mathworks, Natick, MA, USA). N represents the number of animals. Representative values are shown as mean ± SEM. The ratio of labeled neurons between groups was compared using pairwise comparisons, with P-value correction for multiple comparisons via the Tukey-Kramer method. Cohen’s d^75^ was calculated as the effect size for pairs with significant differences. Nuclei that were data-deficient due to the presence of starter neurons in the majority of animals within a group were excluded from the analysis.

To explore relationships between input nuclei, Spearman’s correlation coefficient was calculated for the ratio of input neurons between all combinations of nuclei. Additionally, Spearman’s correlation coefficient was calculated between the ratio of input neurons in one nucleus and the ratio of starter neurons in another nucleus. Cluster analysis using Euclidean distance of the coefficients was used to categorize the nuclei. P-values of the correlation coefficients were adjusted using the Benjamini-Hochberg procedure for controlling the false discovery rate (BH-FDR).^76^

P values lower than 0.1 and higher than 0.05 were mentioned as “tendency of difference”, and those lower than 0.05 were mentioned as “significantly different”. The statistical parameters, P-values, and correlation coefficients are listed in supplemental Tables S2–S16.

## Supplemental information

Document S1. Figure S1 and Tables S2-17

Table S1. List of nuclei that contained input neurons, related to Table 1

Data S1. Data and code for producing graphs in Figures 3 to 8

Data S2. Data and code for producing graphs in Figure 9
