# Supplementary material for: Segregated input to thalamic areas that project differently to core and shell auditory cortical fields
Source: iScience. 2025 Jan 1;28(2):111721. doi: 10.1016/j.isci.2024.111721 (PMC11787697; doi:10.1016/j.isci.2024.111721)
Supplement: Document S1. Figure S1 and Tables S2-17 [file mmc1.pdf]

iScience, Volume *28*

Supplemental information

Segregated input to thalamic areas that project differently to core and shell auditory cortical fields

Tetsufumi Ito (伊藤哲史), Mamiko Yamamoto (山本真未子), Li Liu (劉麗), Khaleeq Ahmad Saqib, Takafumi Furuyama (古山貴文), and Munenori Ono (小野宗範)

Supplemental Information for Ito et al.

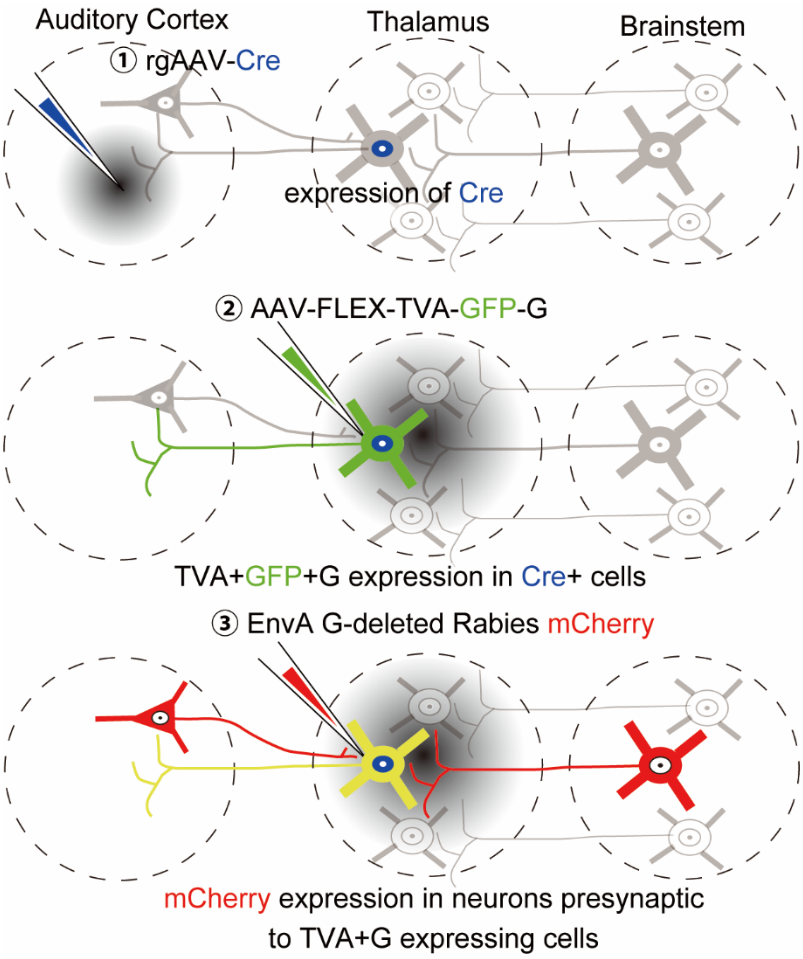

Figure S1, related to Fig. 1 and 2: Schematic of the TRIO experiment. Top: After identifying the auditory areas, rgAAV-Cre was injected into the sound-activated region, leading to Cre recombinase expression in thalamic neurons that project to the injection site. **Middle:** Injection of AAV-FLEX-TVA-GFP-G into the thalamus enabled the expression of TVA (receptor for EnvA-coated virus) and G (glycoprotein necessary for transsynaptic spread of the recombinant rabies virus) in Cre-expressing neurons. eGFP was also expressed in Cre-positive neurons, allowing for fluorescent identification. **Bottom:** Two weeks later, EnvA-coated G-deleted rabies virus (carrying the mCherry gene) was injected into the thalamus. Only neurons expressing both TVA and G (which also express eGFP) were infected by the rabies virus, leading to mCherry expression. These neurons, which express both GFP and mCherry, were identified as the "starter neurons" for monosynaptic tracing. Neurons making synaptic contact with the starter neurons were infected by the rabies virus produced in the starter neurons, leading

to mCherry expression in these second-order neurons. As the rabies virus lacked the G gene, further transsynaptic spread was prevented.

Table S2, related to Figure 3B. Statistical parameters.

Starter nucleus	group 1	group2	Difference	StdErr	pValue	Lower	Upper
BIC	caudal	rostral	-0.021052632	0.04147288	0.868789234	-0.128777206	0.086671943
BIC	caudal	ventral	-0.1346633	0.039405299	0.010042695	-0.237017393	-0.032309206
BIC	rostral	ventral	-0.113610668	0.042888742	0.045349947	-0.225012902	-0.002208434
DLG	caudal	rostral	-0.08005148	0.034346938	0.08199345	-0.169266632	0.009163672
DLG	caudal	ventral	-0.006851852	0.032634612	0.976030237	-0.091619289	0.077915585
DLG	rostral	ventral	0.073199628	0.035519525	0.132042217	-0.019061282	0.165460539
Eth/REth	caudal	rostral	-0.040781965	0.05167093	0.715089735	-0.174995672	0.093431743
Eth/REth	caudal	ventral	0.030717813	0.049094938	0.808482952	-0.09680483	0.158240455
Eth/REth	rostral	ventral	0.071499777	0.053434949	0.396878954	-0.067295915	0.21029547
IGL/PrG	caudal	rostral	-0.032	0.021714063	0.330752617	-0.088401634	0.024401634
IGL/PrG	caudal	ventral	-0.002222222	0.020631534	0.993627087	-0.055812019	0.051367575
IGL/PrG	rostral	ventral	0.029777778	0.02245537	0.403067109	-0.028549378	0.088104933
LP/LD	caudal	rostral	-0.033665244	0.024563546	0.380387708	-0.097468327	0.030137838
LP/LD	caudal	ventral	-0.023809524	0.02333896	0.575981585	-0.084431779	0.036812732
LP/LD	rostral	ventral	0.00985572	0.025402133	0.920803115	-0.056125565	0.075837006
MGD	caudal	rostral	-0.093816287	0.072499062	0.419876165	-0.282130447	0.094497873
MGD	caudal	ventral	-0.128833944	0.068884707	0.18150255	-0.307759925	0.050092037
MGD	rostral	ventral	-0.035017657	0.074974141	0.887580152	-0.229760762	0.159725449
MGM/SG	caudal	rostral	-0.046207728	0.046260469	0.588775085	-0.166367922	0.073952467
MGM/SG	caudal	ventral	-0.027804406	0.043954208	0.804740664	-0.141974157	0.086365345
MGM/SG	rostral	ventral	0.018403322	0.047839777	0.922084024	-0.105859078	0.142665722
MGV	caudal	rostral	-0.091768168	0.052239184	0.217545627	-0.227457896	0.043921561
MGV	caudal	ventral	-0.027676398	0.049634861	0.844199104	-0.156601476	0.101248681
MGV	rostral	ventral	0.06409177	0.054022602	0.478871196	-0.076230334	0.204413874
mRt/p1Rt	caudal	rostral	-0.004	0.019900784	0.978007004	-0.055691696	0.047691696
mRt/p1Rt	caudal	ventral	-0.023809524	0.018908654	0.438562156	-0.072924191	0.025305144
mRt/p1Rt	rostral	ventral	-0.019809524	0.020580186	0.610676969	-0.073265947	0.033646899
Po	caudal	rostral	-0.145182328	0.048343882	0.022849817	-0.270754127	-0.01961053
Po	caudal	ventral	0.011386463	0.045933755	0.966767445	-0.107925103	0.130698029
Po	rostral	ventral	0.156568791	0.049994317	0.017732037	0.026710038	0.286427544
PP/PIL	caudal	rostral	0.577713843	0.11080056	0.000291326	0.289912678	0.865515009
PP/PIL	caudal	ventral	0.49823497	0.10527673	0.00073472	0.22478179	0.77168815
PP/PIL	rostral	ventral	-0.079478873	0.114583233	0.770747881	-0.377105418	0.218147671
SN/VTA	caudal	rostral	0.017857143	0.018346625	0.60417155	-0.02979767	0.065511956
SN/VTA	caudal	ventral	0.011190476	0.017431976	0.799619001	-0.034088563	0.056469515
SN/VTA	rostral	ventral	-0.006666667	0.018972969	0.934500124	-0.05594839	0.042615056
subB	caudal	rostral	-0.029320276	0.054905822	0.855993034	-0.171936519	0.113295966
subB	caudal	ventral	-0.220521298	0.052168557	0.001987817	-0.356027577	-0.085015019
subB	rostral	ventral	-0.191201021	0.056780278	0.011097895	-0.338686107	-0.043715936
ZI	caudal	rostral	0.013491238	0.031131786	0.902319872	-0.067372651	0.094355128
ZI	caudal	ventral	-0.0001258	0.029579748	0.999990028	-0.076958316	0.076706716
ZI	rostral	ventral	-0.013617039	0.032194608	0.906697665	-0.097241578	0.070007501

Table S3, related to Figure 5A. Statistical parameters.

Input nucleus	Difference	StdErr	pValue	Lower	Upper
APT	-0.016916744	0.010405354	0.202470364	-0.050031223	0.016197735
Au1	0.082882238	0.034089682	0.09322206	-0.025606345	0.191370821
AuD	0.016702544	0.011557651	0.244170556	-0.020079058	0.053484147
AuV	0.054275759	0.033673121	0.205387915	-0.05288714	0.161438658
CPu/GP/EP	-0.197058711	0.14061869	0.255639693	-0.644570142	0.250452721
CeA/BNST/EA	0.000210832	0.003580708	0.95675039	-0.011184579	0.011606243
Ect/PRh	0.00989712	0.014856269	0.552939717	-0.037382159	0.0571764
Insura	0.0025301	0.001679312	0.228999034	-0.00281422	0.00787442
PAG	-0.009594015	0.003749127	0.083287098	-0.021525411	0.002337381
PaF/SPF	-0.017529978	0.000365906	2.38E-05	-0.018694456	-0.016365501
RTN	0.056827963	0.020864408	0.0723251	-0.009571895	0.123227822
S1/S2	0.01696708	0.01051069	0.204876334	-0.016482628	0.050416789
V1/V2	0.002227588	0.002367697	0.416201949	-0.005307479	0.009762655
c IC cortex	0.0076075	0.003520983	0.119506466	-0.00359784	0.018812839
i DLL	-0.010085565	0.011071442	0.429485053	-0.045319836	0.025148706
i IC cortex	0.027169975	0.062081056	0.691231257	-0.170399651	0.224739601
i ICC	0.155770164	0.115533347	0.270332099	-0.211908509	0.523448838
i VLL/ILL/PL	-4.09E-05	0.003048591	0.990130786	-0.009742909	0.009661047
i/c SC	0.014053359	0.027758733	0.647529874	-0.07428732	0.102394038
i/c hypothal	-0.005705982	0.002144288	0.07627297	-0.012530062	0.001118098

Table S4, related to Figure 5B. Statistical parameters.

Input nucleus	group 1	group 2	Difference	StdErr	pValue	Lower	Upper
APT	caudal	core	0.0105	0.0090	0.6628	-0.0177	0.0387
APT	caudal	rostral	-0.0063	0.0084	0.8719	-0.0324	0.0198
APT	caudal	ventral	0.0063	0.0102	0.9238	-0.0256	0.0383
APT	core	rostral	-0.0168	0.0090	0.3074	-0.0450	0.0114
APT	core	ventral	-0.0042	0.0108	0.9791	-0.0379	0.0295
APT	rostral	ventral	0.0126	0.0102	0.6219	-0.0193	0.0446
Au1	caudal	core	-0.0795	0.0178	0.0070	-0.1350	-0.0239
Au1	caudal	rostral	-0.0175	0.0165	0.7198	-0.0690	0.0340
Au1	caudal	ventral	-0.0009	0.0202	1.0000	-0.0640	0.0621
Au1	core	rostral	0.0620	0.0178	0.0293	0.0064	0.1175
Au1	core	ventral	0.0785	0.0213	0.0214	0.0121	0.1450
Au1	rostral	ventral	0.0166	0.0202	0.8434	-0.0465	0.0796
AuD	caudal	core	-0.0179	0.0111	0.4196	-0.0525	0.0168
AuD	caudal	rostral	-0.0185	0.0103	0.3320	-0.0506	0.0136
AuD	caudal	ventral	-0.0028	0.0126	0.9957	-0.0421	0.0365
AuD	core	rostral	-0.0006	0.0111	0.9999	-0.0353	0.0340
AuD	core	ventral	0.0150	0.0133	0.6793	-0.0264	0.0565
AuD	rostral	ventral	0.0157	0.0126	0.6152	-0.0236	0.0550
AuV	caudal	core	-0.0550	0.0168	0.0396	-0.1074	-0.0026
AuV	caudal	rostral	-0.0079	0.0155	0.9552	-0.0564	0.0406
AuV	caudal	ventral	-0.0060	0.0190	0.9886	-0.0654	0.0534
AuV	core	rostral	0.0471	0.0168	0.0803	-0.0053	0.0995
AuV	core	ventral	0.0490	0.0201	0.1373	-0.0136	0.1116
AuV	rostral	ventral	0.0019	0.0190	0.9996	-0.0575	0.0613
c IC cortex	caudal	core	-0.0056	0.0047	0.6446	-0.0203	0.0091
c IC cortex	caudal	rostral	-0.0029	0.0044	0.9071	-0.0165	0.0107
c IC cortex	caudal	ventral	-0.0166	0.0053	0.0508	-0.0333	0.0001
c IC cortex	core	rostral	0.0027	0.0047	0.9361	-0.0120	0.0174
c IC cortex	core	ventral	-0.0110	0.0056	0.2738	-0.0286	0.0066
c IC cortex	rostral	ventral	-0.0137	0.0053	0.1146	-0.0304	0.0030
CeA/BNST/EA	caudal	core	0.0088	0.0065	0.5571	-0.0115	0.0291
CeA/BNST/EA	caudal	rostral	0.0101	0.0060	0.3856	-0.0087	0.0289
CeA/BNST/EA	caudal	ventral	0.0048	0.0074	0.9131	-0.0182	0.0278
CeA/BNST/EA	core	rostral	0.0013	0.0065	0.9966	-0.0190	0.0217
CeA/BNST/EA	core	ventral	-0.0040	0.0078	0.9538	-0.0283	0.0203
CeA/BNST/EA	rostral	ventral	-0.0053	0.0074	0.8852	-0.0284	0.0177
CPu/GP/EP	caudal	core	0.1287	0.0845	0.4645	-0.1352	0.3926
CPu/GP/EP	caudal	rostral	0.1079	0.0783	0.5416	-0.1365	0.3522
CPu/GP/EP	caudal	ventral	0.1061	0.0959	0.6947	-0.1932	0.4053
CPu/GP/EP	core	rostral	-0.0208	0.0845	0.9944	-0.2847	0.2431
CPu/GP/EP	core	ventral	-0.0226	0.1010	0.9958	-0.3380	0.2928
CPu/GP/EP	rostral	ventral	-0.0018	0.0959	1.0000	-0.3010	0.2975
Ect/PRh	caudal	core	-0.0113	0.0097	0.6632	-0.0416	0.0190
Ect/PRh	caudal	rostral	-0.0016	0.0090	0.9978	-0.0297	0.0265
Ect/PRh	caudal	ventral	-0.0173	0.0110	0.4379	-0.0517	0.0171
Ect/PRh	core	rostral	0.0097	0.0097	0.7551	-0.0206	0.0400
Ect/PRh	core	ventral	-0.0060	0.0116	0.9519	-0.0423	0.0302
Ect/PRh	rostral	ventral	-0.0157	0.0110	0.5143	-0.0501	0.0187
i DLL	caudal	core	0.0048	0.0062	0.8597	-0.0144	0.0241
i DLL	caudal	rostral	0.0128	0.0057	0.1837	-0.0050	0.0307
i DLL	caudal	ventral	-0.0035	0.0070	0.9571	-0.0254	0.0184
i DLL	core	rostral	0.0080	0.0062	0.5903	-0.0113	0.0272
i DLL	core	ventral	-0.0083	0.0074	0.6814	-0.0314	0.0147
i DLL	rostral	ventral	-0.0163	0.0070	0.1620	-0.0382	0.0055
i IC cortex	caudal	core	0.0120	0.0583	0.9967	-0.1699	0.1938
i IC cortex	caudal	rostral	-0.0038	0.0539	0.9999	-0.1722	0.1646
i IC cortex	caudal	ventral	-0.1938	0.0661	0.0663	-0.4000	0.0124
i IC cortex	core	rostral	-0.0158	0.0583	0.9926	-0.1976	0.1661
i IC cortex	core	ventral	-0.2058	0.0696	0.0642	-0.4231	0.0116
i IC cortex	rostral	ventral	-0.1900	0.0661	0.0723	-0.3962	0.0162

Input nucleus	group 1	group 2	Difference	StdErr	pValue	Lower	Upper
i ICC	caudal	core	-0.1483	0.0715	0.2318	-0.3714	0.0748
i ICC	caudal	rostral	-0.0966	0.0662	0.4967	-0.3032	0.1099
i ICC	caudal	ventral	-0.0215	0.0810	0.9930	-0.2744	0.2315
i ICC	core	rostral	0.0517	0.0715	0.8856	-0.1714	0.2747
i ICC	core	ventral	0.1268	0.0854	0.4838	-0.1398	0.3934
i ICC	rostral	ventral	0.0752	0.0810	0.7913	-0.1778	0.3281
i VLL/ILL/PL	caudal	core	0.0017	0.0048	0.9836	-0.0133	0.0167
i VLL/ILL/PL	caudal	rostral	-0.0035	0.0044	0.8545	-0.0174	0.0103
i VLL/ILL/PL	caudal	ventral	-0.0159	0.0054	0.0666	-0.0329	0.0010
i VLL/ILL/PL	core	rostral	-0.0052	0.0048	0.7026	-0.0202	0.0097
i VLL/ILL/PL	core	ventral	-0.0176	0.0057	0.0534	-0.0355	0.0003
i VLL/ILL/PL	rostral	ventral	-0.0124	0.0054	0.1737	-0.0293	0.0046
i/c hypothal	caudal	core	0.0135	0.0088	0.4600	-0.0141	0.0411
i/c hypothal	caudal	rostral	0.0040	0.0082	0.9596	-0.0216	0.0296
i/c hypothal	caudal	ventral	0.0065	0.0100	0.9145	-0.0248	0.0378
i/c hypothal	core	rostral	-0.0095	0.0088	0.7107	-0.0371	0.0181
i/c hypothal	core	ventral	-0.0071	0.0106	0.9064	-0.0401	0.0259
i/c hypothal	rostral	ventral	0.0025	0.0100	0.9944	-0.0288	0.0338
i/c SC	caudal	core	0.0024	0.0219	0.9995	-0.0661	0.0708
i/c SC	caudal	rostral	-0.0132	0.0203	0.9135	-0.0765	0.0502
i/c SC	caudal	ventral	-0.0966	0.0249	0.0160	-0.1741	-0.0190
i/c SC	core	rostral	-0.0155	0.0219	0.8916	-0.0839	0.0529
i/c SC	core	ventral	-0.0989	0.0262	0.0188	-0.1807	-0.0171
i/c SC	rostral	ventral	-0.0834	0.0249	0.0352	-0.1610	-0.0058
Insura	caudal	core	-0.0020	0.0062	0.9873	-0.0212	0.0172
Insura	caudal	rostral	-0.0121	0.0057	0.2168	-0.0299	0.0057
Insura	caudal	ventral	-0.0012	0.0070	0.9979	-0.0230	0.0205
Insura	core	rostral	-0.0101	0.0062	0.4049	-0.0293	0.0091
Insura	core	ventral	0.0008	0.0074	0.9996	-0.0222	0.0237
Insura	rostral	ventral	0.0109	0.0070	0.4462	-0.0109	0.0326
PaF/SPF	caudal	core	0.0179	0.0068	0.1076	-0.0035	0.0392
PaF/SPF	caudal	rostral	0.0057	0.0063	0.8067	-0.0141	0.0255
PaF/SPF	caudal	ventral	0.0029	0.0078	0.9809	-0.0213	0.0271
PaF/SPF	core	rostral	-0.0122	0.0068	0.3421	-0.0335	0.0092
PaF/SPF	core	ventral	-0.0149	0.0082	0.3218	-0.0405	0.0106
PaF/SPF	rostral	ventral	-0.0028	0.0078	0.9834	-0.0270	0.0214
PAG	caudal	core	0.0095	0.0064	0.4822	-0.0104	0.0295
PAG	caudal	rostral	0.0039	0.0059	0.9101	-0.0146	0.0224
PAG	caudal	ventral	0.0039	0.0072	0.9458	-0.0187	0.0266
PAG	core	rostral	-0.0056	0.0064	0.8158	-0.0256	0.0143
PAG	core	ventral	-0.0056	0.0076	0.8833	-0.0294	0.0183
PAG	rostral	ventral	0.0001	0.0072	1.0000	-0.0226	0.0227
RTN	caudal	core	-0.0535	0.0209	0.1156	-0.1186	0.0117
RTN	caudal	rostral	-0.0308	0.0193	0.4279	-0.0911	0.0295
RTN	caudal	ventral	-0.0116	0.0237	0.9598	-0.0854	0.0623
RTN	core	rostral	0.0227	0.0209	0.7065	-0.0425	0.0878
RTN	core	ventral	0.0419	0.0249	0.3866	-0.0360	0.1198
RTN	rostral	ventral	0.0193	0.0237	0.8467	-0.0546	0.0931
S1/S2	caudal	core	-0.0151	0.0061	0.1301	-0.0342	0.0039
S1/S2	caudal	rostral	-0.0063	0.0057	0.6925	-0.0239	0.0114
S1/S2	caudal	ventral	0.0012	0.0069	0.9979	-0.0204	0.0228
S1/S2	core	rostral	0.0089	0.0061	0.5007	-0.0102	0.0279
S1/S2	core	ventral	0.0164	0.0073	0.1831	-0.0064	0.0392
S1/S2	rostral	ventral	0.0075	0.0069	0.7073	-0.0141	0.0291
V1/V2	caudal	core	-0.0012	0.0166	0.9999	-0.0529	0.0506
V1/V2	caudal	rostral	-0.0230	0.0153	0.4761	-0.0709	0.0249
V1/V2	caudal	ventral	-0.0283	0.0188	0.4720	-0.0870	0.0303
V1/V2	core	rostral	-0.0218	0.0166	0.5752	-0.0736	0.0299
V1/V2	core	ventral	-0.0272	0.0198	0.5455	-0.0890	0.0347
V1/V2	rostral	ventral	-0.0053	0.0188	0.9915	-0.0640	0.0533

Table S5, related to Figure 6. Statistical parameters.

IC cortex						
group	P value	Lower	Upper	t	df	cohen's d
core	0.0025	0.7026	0.9841	6.7733	4	3.0291
rostral	0.0014	0.7282	0.9735	7.9409	4	3.5513
ventral	0.0254	0.5468	0.9629	3.1491	5	1.2856
caudal	0.3664	0.2579	1.0468	0.9928	5	0.4053
ICC						
group	P value	Lower	Upper	t	df	cohen's d
core	0.0447	0.5108	1.0516	2.8869	4	1.291
rostral	0.5954	-0.0728	1.3347	0.5922	3	0.2961
ventral	0.4596	0.1684	1.0718	0.8462	3	0.4231
caudal	0.0728	0.4108	1.367	3.5	2	2.0207

Table S6, related to Figure 7A. Correlation coefficients between every combination of input nuclei.

	Au1	Insura	APT	AuD	AuV	CPu/GP/EP	CeA/BNST/EA	i DLL	Ect/PRh	i IC cortex	c IC cortex	i ICC	i VLL/ILL/PL	PAG	PaF/SPF	RTN	S1/S2	i/c SC	V1/V2	i/c hypothal
Au1	1	0.487770683	-0.429014342	0.688926179	0.783436619	-0.591286806	-0.325366486	-0.432181163	0.1200984	0.054545776	0.078150798	0.77003583	-0.36217095	-0.514616817	-0.353880022	0.803008735	0.808475509	-0.177588095	0.330385054	-0.622030981
Insura	0.487770683	1	-0.302658735	0.502148685	0.59568522	-0.431645364	-0.002695764	-0.339146382	-0.16140819	0.153174654	0.238289678	0.595476943	-0.18711891	-0.297020648	-0.350657164	0.520717862	0.488702202	-0.012800894	0.316494971	-0.503664474
APT	-0.429014342	-0.302658735	1	-0.327660187	-0.39379845	0.186863716	-0.240667383	-0.00995541	-0.072601377	-0.404636221	-0.440381113	-0.617842401	-0.139555287	0.205525195	0.469072857	-0.506940191	-0.282616179	-0.015444131	0.245588424	0.378947368
AuD	0.688926179	0.502148685	-0.327660187	1	0.738852674	-0.53002631	-0.19840725	-0.158486481	0.246558684	0.306507299	0.322066806	0.750343264	-0.286497753	-0.412772686	-0.178071067	0.766920729	0.586425396	-0.045857589	0.520543051	-0.472852932
AuV	0.783436619	0.59568522	-0.39379845	0.738852674	1	-0.412637145	-0.159195812	-0.229697059	0.202673876	0.15202339	0.365627893	0.707390898	-0.280958372	-0.43647003	-0.305988194	0.626779801	0.61801913	-0.003095499	0.492319583	-0.529768815
CPu/GP/EP	-0.591286806	-0.431645364	0.186863716	-0.53002631	-0.412637145	1	0.345369212	0.439569029	0.051578152	-0.010869565	-0.042595363	-0.668461538	0.347258617	0.444700912	0.495756591	-0.602421565	-0.706568973	0.326086957	-0.09448426	0.716607658
CeA/BNST/EA	-0.325366486	-0.002695764	-0.240667383	-0.19840725	-0.159195812	0.345369212	1	0.168063637	-0.021016691	0.211124165	0.347900405	-0.2253156	0.256073952	0.311607606	0.0050418	-0.181661785	-0.228818705	0.303314869	-0.042563555	0.608293463
i DLL	-0.432181163	-0.339146382	-0.00995541	-0.158486481	-0.229697059	0.439569029	0.168063637	1	-0.022465753	0.053146009	0.205669129	-0.407097142	0.018948655	0.317436867	0.323617233	-0.261790227	-0.484102065	0.352683514	-0.175556517	0.259804702
Ect/PRh	0.1200984	-0.16140819	-0.072601377	0.246558684	0.202673876	0.051578152	-0.021016691	-0.022465753	1	0.195347292	0.01568318	0.134173076	0.363916467	0.081610304	0.143328188	0.351491878	0.097675947	0.158827152	0.388943544	0.088235559
i IC cortex	0.054545776	0.153174654	-0.404636221	0.306507299	0.15202339	-0.010869565	0.211124165	0.053146009	0.195347292	1	0.605808952	0.288156288	0.503464352	0.032972066	0.084507438	0.161106998	0.183829565	0.541880342	0.08720587	-0.028571642
c IC cortex	0.078150798	0.238289678	-0.440381113	0.322066806	0.365627893	-0.042595363	0.347900405	0.205669129	0.01568318	0.605808952	1	0.302538395	0.238909508	0.063211841	-0.056788691	0.194427142	0.038136646	0.595084575	0.300093117	0.018162277
i ICC	0.77003583	0.595476943	-0.617842401	0.750343264	0.707390898	-0.668461538	-0.2253156	-0.407097142	0.134173076	0.288156288	0.302538395	1	-0.131948711	-0.441433677	-0.425186481	0.730909804	0.623629839	-0.105090312	0.216039678	-0.678508998
i VLL/ILL/PL	-0.36217095	-0.18711891	-0.139555287	-0.286497753	-0.280958372	0.347258617	0.256073952	0.018948655	0.363916467	0.503464352	0.238909508	-0.131948711	1	0.443561983	0.300246	-0.168879253	-0.278799857	0.298665824	-0.013924334	0.31685667
PAG	-0.514616817	-0.297020648	0.205525195	-0.412772686	-0.43647003	0.444700912	0.311607606	0.317436867	0.081610304	0.032972066	0.063211841	-0.441433677	0.443561983	1	0.255834758	-0.30934975	-0.386445773	0.391405505	-0.162672027	0.632677612
PaF/SPF	-0.353880022	-0.350657164	0.469072857	-0.178071067	-0.305988194	0.495756591	0.0050418	0.323617233	0.143328188	0.084507438	-0.056788691	-0.425186481	0.300246	0.255834758	1	-0.5140023	-0.492499302	0.300769519	0.086330097	0.499385749
RTN	0.803008735	0.520717862	-0.506940191	0.766920729	0.626779801	-0.602421565	-0.181661785	-0.261790227	0.351491878	0.161106998	0.194427142	0.730909804	-0.168879253	-0.30934975	-0.5140023	1	0.725241413	-0.164383566	0.372177612	-0.514090471
S1/S2	0.808475509	0.488702202	-0.282616179	0.586425396	0.61801913	-0.706568973	-0.228818705	-0.484102065	0.097675947	0.183829565	0.038136646	0.623629839	-0.278799857	-0.386445773	-0.492499302	0.725241413	1	-0.18479521	0.316841337	-0.611052883
i/c SC	-0.177588095	-0.012800894	-0.015444131	-0.045857589	-0.003095499	0.326086957	0.303314869	0.352683514	0.158827152	0.541880342	0.595084575	-0.105090312	0.298665824	0.391405505	0.300769519	-0.164383566	-0.18479521	1	0.19741091	0.393484305
V1/V2	0.330385054	0.316494971	0.245588424	0.520543051	0.492319583	-0.09448426	-0.042563555	-0.175556517	0.388943544	0.08720587	0.300093117	0.216039678	-0.013924334	-0.162672027	0.086330097	0.372177612	0.316841337	0.19741091	1	0.054903449
i/c hypothal	-0.622030981	-0.503664474	0.378947368	-0.472852932	-0.529768815	0.716607658	0.608293463	0.259804702	0.088235559	-0.028571642	0.018162277	-0.678508998	0.31685667	0.632677612	0.499385749	-0.514090471	-0.611052883	0.393484305	0.054903449	1

Table S7, related to Figure 7A. P-values of correlation coefficients between every combination of input nuclei.

	Au1	Insura	APT	AuD	AuV	CPu/GP/I	CeA/BNS	i DLL	Ect/PRh	i IC cortex	c IC corte	i ICC	i VLL/ILL/	PAG	PaF/SPF	RTN	S1/S2	i/c SC	V1/V2	i/c hypoth
Au1	1	0.038994	0.10272	0.000916	0.000116	0.015669	0.212684	0.078667	0.710602	0.932165	0.844672	0.000116	0.174775	0.03079	0.173919	0.000116	3.8880741	0.548202	0.209166	0.006696
Insura	0.038994	1	0.255525	0.03692	0.007483	0.100636	1	0.179734	0.580142	0.602159	0.370786	0.006696	0.528873	0.241216	0.172727	0.038009	0.038009	1	0.212684	0.038009
APT	0.10272	0.255525	1	0.237997	0.146997	0.574505	0.393811	1	0.885074	0.132592	0.085441	0.006696	0.669415	0.486774	0.062535	0.062922	0.296958	1	0.387261	0.173919
AuD	0.000916	0.03692	0.237997	1	0.000465	0.038009	0.486774	0.58815	0.370786	0.251167	0.212684	0.000171	0.291423	0.095199	0.53622	0.000411	0.007483	0.953866	0.028212	0.060028
AuV	0.000116	0.007483	0.146997	0.000465	1	0.132592	0.589306	0.408348	0.486774	0.615393	0.171057	0.00082	0.315789	0.081558	0.241216	0.007483	0.006578	1	0.040399	0.031639
CPu/GP/I	0.015669	0.100636	0.574505	0.038009	0.132592	1	0.212684	0.09459	0.943359	1	0.964902	0.003464	0.214206	0.089396	0.048873	0.015669	0.001213	0.241216	0.8253	0.000931
CeA/BNS	0.212684	1	0.393811	0.486774	0.589306	0.212684	1	0.553681	1	0.464748	0.173919	0.396569	0.349217	0.219484	1	0.553681	0.390756	0.241216	0.955459	0.006696
i DLL	0.078667	0.179734	1	0.58815	0.408348	0.09459	0.553681	1	1	0.932165	0.458818	0.094651	1	0.212684	0.209166	0.361729	0.038009	0.173919	0.53622	0.340414
Ect/PRh	0.710602	0.580142	0.885074	0.370786	0.486774	0.943359	1	1	1	0.521379	1	0.652716	0.173919	0.834015	0.618345	0.209166	0.791047	0.589306	0.123622	0.82654
i IC cortex	0.932165	0.602159	0.132592	0.251167	0.615393	1	0.464748	0.932165	0.521379	1	0.006696	0.273503	0.043823	0.988988	0.833522	0.616962	0.535359	0.027386	0.82654	1
c IC corte	0.844672	0.370786	0.085441	0.212684	0.171057	0.964902	0.173919	0.458818	1	0.006696	1	0.237997	0.389972	0.897117	0.921949	0.528873	0.964902	0.006696	0.241216	1
i ICC	0.000116	0.006696	0.006696	0.000171	0.00082	0.003464	0.396569	0.094651	0.652716	0.273503	0.237997	1	0.669	0.063538	0.078667	0.00082	0.003763	0.762283	0.423316	0.001432
i VLL/ILL/	0.174775	0.528873	0.669415	0.291423	0.315789	0.214206	0.349217	1	0.173919	0.043823	0.389972	0.669	1	0.077274	0.251167	0.589306	0.294555	0.263468	1	0.241216
PAG	0.03079	0.241216	0.486774	0.095199	0.081558	0.089396	0.219484	0.212684	0.834015	0.988988	0.897117	0.063538	0.077274	1	0.325042	0.255525	0.119942	0.121317	0.570231	0.004686
PaF/SPF	0.173919	0.172727	0.062535	0.53622	0.241216	0.048873	1	0.209166	0.618345	0.833522	0.921949	0.078667	0.251167	0.325042	1	0.038994	0.03692	0.241216	0.8253	0.038104
RTN	0.000116	0.038009	0.062922	0.000411	0.007483	0.015669	0.553681	0.361729	0.209166	0.616962	0.528873	0.00082	0.589306	0.255525	0.038994	1	0.000821	0.602159	0.173919	0.043823
S1/S2	3.8880741	0.038009	0.296958	0.007483	0.006578	0.001213	0.390756	0.038009	0.791047	0.535359	0.964902	0.003763	0.294555	0.119942	0.03692	0.000821	1	0.528873	0.212684	0.006696
i/c SC	0.548202	1	1	0.953866	1	0.241216	0.241216	0.173919	0.589306	0.027386	0.006696	0.762283	0.263468	0.121317	0.241216	0.602159	0.528873	1	0.486774	0.135442
V1/V2	0.209166	0.212684	0.387261	0.028212	0.040399	0.8253	0.955459	0.53622	0.123622	0.82654	0.241216	0.423316	1	0.570231	0.8253	0.173919	0.212684	0.486774	1	0.932165
i/c hypoth	0.006696	0.038009	0.173919	0.060028	0.031639	0.000931	0.006696	0.340414	0.82654	1	1	0.001432	0.241216	0.004686	0.038104	0.043823	0.006696	0.135442	0.932165	1

Table S8, related to Figure 7B. Correlation coefficients between every combination of input and stater nuclei.

	BIC	DLG	Eth/REth	IGL/PrG	LP/LD	MGV	MGD	MGM/SG	PP/PIL	Po	SN/VTA	ZI	subB	mRt/p1Rt
Au1	-0.1612	0.0284	-0.0350	0.2145	-0.0712	0.6835	0.3120	-0.0924	-0.4202	0.2302	-0.3148	0.0547	-0.2007	-0.1318
Insura	0.0425	0.0923	-0.0200	-0.0189	0.3372	0.4022	0.5335	0.3269	-0.5043	0.3953	0.0980	0.2672	-0.0814	0.3206
APT	-0.1427	0.2802	0.2684	0.1122	0.1952	-0.6344	-0.1690	0.0360	0.3429	-0.0215	0.0995	-0.1386	0.0231	-0.0735
AuD	0.1239	0.0918	-0.4081	0.1600	0.1103	0.6059	0.4303	-0.0264	-0.2916	-0.1688	-0.2776	-0.0008	-0.2529	-0.1817
AuV	0.1192	0.1301	-0.1691	0.1970	-0.0104	0.6800	0.3940	-0.1360	-0.3901	0.1342	-0.1486	0.2529	0.0412	-0.1108
CPu/GP/EP	0.0195	-0.1205	-0.1329	-0.0976	-0.4831	-0.3915	-0.2901	-0.0012	0.5155	-0.0111	0.2092	0.1616	0.4483	-0.0849
CeA/BNST/EA	0.0843	-0.0818	-0.0163	0.1667	-0.2139	-0.1801	0.0359	0.1171	0.1542	-0.0219	0.3577	0.1560	0.1692	0.4514
i DLL	0.1909	-0.1340	-0.3418	0.0453	-0.2770	-0.2173	-0.0739	-0.3302	0.3775	-0.4330	0.0154	-0.1220	0.2056	-0.1151
Ect/PRh	-0.0383	-0.0562	0.0472	0.1910	-0.1390	0.2622	0.0151	-0.1701	0.0083	-0.1690	-0.1900	-0.1639	-0.1531	-0.2305
i IC cortex	0.5887	0.0128	-0.1636	0.1252	-0.0030	0.2888	0.3096	0.3572	-0.2885	-0.1867	0.0990	-0.2906	0.4394	-0.0464
c IC cortex	0.4912	0.1691	-0.2314	0.1460	-0.0924	0.2911	0.3732	-0.0183	-0.3276	-0.0977	0.1263	0.0543	0.4102	0.1212
i ICC	0.1911	0.0591	-0.0766	0.0258	0.2407	0.7312	0.3129	0.0917	-0.5317	0.1644	-0.1793	0.1348	-0.1989	-0.1551
i VLL/ILL/PL	0.4842	-0.0968	0.3931	-0.0524	-0.0909	-0.1238	-0.1410	0.3561	-0.0289	0.0666	0.1063	-0.2093	0.2498	-0.0067
PAG	0.1625	-0.1113	0.2841	0.1377	-0.1158	-0.4730	-0.2218	0.0821	0.3656	0.0395	-0.1528	-0.0749	0.2591	0.0764
PaF/SPF	0.0741	0.2652	0.1972	-0.0476	-0.1746	-0.3164	-0.2108	-0.1115	0.4772	0.0582	-0.0803	-0.2102	0.2849	-0.3162
RTN	-0.0831	0.0024	-0.2572	0.1613	-0.0096	0.5499	0.3466	0.0575	-0.4397	-0.0561	-0.3449	0.0335	-0.4043	-0.0736
S1/S2	-0.1313	-0.0784	0.0288	0.3881	-0.0190	0.5045	0.2708	0.0791	-0.3773	0.0761	-0.0898	-0.0767	-0.2652	0.0204
i/c SC	0.3113	0.1997	0.0576	0.3237	-0.0270	-0.1328	0.3877	0.0731	-0.1273	-0.0488	-0.0948	-0.0838	0.6894	0.0255
V1/V2	0.0340	0.2191	-0.0482	0.4211	-0.0241	0.1482	0.3675	0.0985	-0.1413	-0.0716	0.0937	-0.1376	0.0988	0.0478
i/c hypothal	0.0073	0.0792	0.1087	0.0307	-0.3507	-0.6541	-0.2564	0.1046	0.5394	-0.1456	0.1860	0.1046	0.3635	0.0253

Table S9, related to Figure 7B. P-values of correlation coefficients between every combination of input and stater nuclei.

	BIC	DLG	Eth/REth	IGL/PrG	LP/LD	MGV	MGD	MGM/SG	PP/PIL	Po	SN/VTA	ZI	subB	mRt/p1Rt
Au1	0.8739	0.9750	0.9750	0.7664	0.9180	0.0057	0.4602	0.9117	0.2690	0.7185	0.4570	0.9599	0.8140	0.8940
Insura	0.9686	0.9117	0.9750	0.9750	0.4046	0.2808	0.0839	0.4329	0.1056	0.2959	0.9117	0.5726	0.9117	0.4349
APT	0.8940	0.5726	0.6163	0.9117	0.8408	0.0233	0.8739	0.9750	0.4349	0.9750	0.9117	0.8940	0.9750	0.9180
AuD	0.9004	0.9117	0.2808	0.8739	0.9117	0.0253	0.2565	0.9750	0.5410	0.8739	0.5700	0.9968	0.6396	0.8421
AuV	0.9064	0.9004	0.8739	0.8267	0.9870	0.0067	0.3150	0.8940	0.3150	0.8940	0.8940	0.6538	0.9745	0.9117
CPu/GP/EP	0.9750	0.9112	0.9004	0.9117	0.1925	0.3369	0.5726	0.9968	0.1459	0.9870	0.8254	0.8794	0.2649	0.9117
CeA/BNST/EA	0.9117	0.9117	0.9775	0.8739	0.7658	0.8421	0.9750	0.9064	0.8739	0.9750	0.3534	0.8739	0.8739	0.1925
i DLL	0.8254	0.8940	0.3972	0.9625	0.5516	0.7510	0.9117	0.4319	0.3150	0.2415	0.9775	0.9004	0.7892	0.9064
Ect/PRh	0.9750	0.9599	0.9625	0.8318	0.8940	0.5994	0.9775	0.8739	0.9876	0.8739	0.8318	0.8739	0.8794	0.7185
i IC cortex	0.0433	0.9845	0.8739	0.9004	0.9968	0.5516	0.4923	0.3972	0.5516	0.8421	0.9117	0.5516	0.2565	0.9625
c IC cortex	0.1271	0.8739	0.6991	0.8872	0.9117	0.5248	0.3152	0.9750	0.4329	0.9117	0.9004	0.9599	0.2690	0.9004
i ICC	0.8254	0.9549	0.9117	0.9750	0.6571	0.0019	0.4441	0.9117	0.0839	0.8739	0.8421	0.8940	0.8140	0.8739
i VLL/ILL/PL	0.1547	0.9117	0.3150	0.9606	0.9117	0.9030	0.8940	0.3972	0.9750	0.9352	0.9117	0.8092	0.6571	0.9876
PAG	0.8739	0.9112	0.5410	0.8940	0.9064	0.1486	0.7375	0.9117	0.3330	0.9745	0.8762	0.9117	0.5965	0.9117
PaF/SPF	0.9117	0.5726	0.8140	0.9606	0.8589	0.4349	0.7664	0.9112	0.1459	0.9552	0.9117	0.7664	0.5410	0.4349
RTN	0.9117	0.9968	0.6676	0.8794	0.9876	0.1027	0.4349	0.9599	0.2690	0.9606	0.4349	0.9750	0.3150	0.9208
S1/S2	0.8940	0.9117	0.9750	0.3150	0.9750	0.1056	0.5723	0.9117	0.3150	0.9117	0.9117	0.9117	0.5726	0.9750
i/c SC	0.4602	0.8140	0.9592	0.4349	0.9750	0.8940	0.3150	0.9145	0.9004	0.9606	0.9117	0.9117	0.0057	0.9750
V1/V2	0.9750	0.7468	0.9606	0.2565	0.9750	0.8794	0.3324	0.9117	0.8940	0.9145	0.9117	0.8940	0.9117	0.9606
i/c hypothal	0.9876	0.9117	0.9117	0.9750	0.4046	0.0120	0.6405	0.9117	0.0939	0.8940	0.8421	0.9117	0.3797	0.9750

Table S10, related to Figure 8A. Statistical parameters.

layer	group 1	group 2	Difference	StdErr	pValue	Lower	Upper
Layer 2-3	caudal	core	0.0183	0.0165	0.6897	-0.0282	0.0647
Layer 2-3	caudal	rostral	0.0056	0.0165	0.9859	-0.0408	0.0521
Layer 2-3	caudal	ventral	0.0193	0.0157	0.6156	-0.0248	0.0634
Layer 2-3	core	rostral	-0.0127	0.0178	0.8922	-0.0628	0.0375
Layer 2-3	core	ventral	0.0010	0.0171	0.9999	-0.0470	0.0491
Layer 2-3	rostral	ventral	0.0137	0.0171	0.8530	-0.0343	0.0617
Layer 4	caudal	core	0.0420	0.0348	0.6308	-0.0560	0.1400
Layer 4	caudal	rostral	0.0104	0.0348	0.9903	-0.0875	0.1084
Layer 4	caudal	ventral	0.0398	0.0331	0.6337	-0.0533	0.1328
Layer 4	core	rostral	-0.0316	0.0376	0.8354	-0.1374	0.0743
Layer 4	core	ventral	-0.0023	0.0360	0.9999	-0.1036	0.0991
Layer 4	rostral	ventral	0.0293	0.0360	0.8473	-0.0720	0.1306
Layer 5	caudal	core	0.4116	0.1580	0.0753	-0.0327	0.8559
Layer 5	caudal	rostral	0.2720	0.1580	0.3406	-0.1723	0.7163
Layer 5	caudal	ventral	0.1696	0.1501	0.6763	-0.2526	0.5918
Layer 5	core	rostral	-0.1396	0.1707	0.8453	-0.6195	0.3403
Layer 5	core	ventral	-0.2420	0.1634	0.4677	-0.7015	0.2175
Layer 5	rostral	ventral	-0.1024	0.1634	0.9222	-0.5619	0.3571
Layer 6	caudal	core	-0.4719	0.1726	0.0584	-0.9572	0.0134
Layer 6	caudal	rostral	-0.2881	0.1726	0.3664	-0.7734	0.1973
Layer 6	caudal	ventral	-0.2287	0.1640	0.5178	-0.6898	0.2325
Layer 6	core	rostral	0.1838	0.1864	0.7590	-0.3404	0.7080
Layer 6	core	ventral	0.2432	0.1785	0.5366	-0.2587	0.7451
Layer 6	rostral	ventral	0.0594	0.1785	0.9869	-0.4425	0.5613

Table S11, related to Figure 8B. Statistical parameters.

layer	Difference	StdErr	pValue	Lower	Upper
Layer 2-3	-0.024434389	0.025825005	0.37557779	-0.085500822	0.036632044
Layer 4	-0.067307692	0.059117024	0.292351998	-0.20709724	0.072481855
Layer 5	-0.579850302	0.212767849	0.029539729	-1.082966317	-0.076734286
Layer 6	0.671592383	0.22258054	0.019460376	0.145273039	1.197911727

Table S12, related to Figure 8C. Statistical parameters.

Input nucleus	group 1	group 2	Difference	StdErr	pValue	Lower	Upper
Au1	caudal	core	-0.285772275	0.093071987	0.029387602	-0.547476393	-0.024068156
Au1	caudal	rostral	-0.076904666	0.093071987	0.841338789	-0.338608785	0.184799453
Au1	caudal	ventral	0.009795705	0.088431994	0.999495528	-0.238861466	0.258452876
Au1	core	rostral	0.208867609	0.100529236	0.196062324	-0.073805147	0.491540364
Au1	core	ventral	0.29556798	0.096249416	0.029362338	0.024929421	0.566206538
Au1	rostral	ventral	0.086700371	0.096249416	0.804536609	-0.183938188	0.35733893
AuD	caudal	core	0.09091697	0.1351623	0.906121554	-0.289138638	0.470972577
AuD	caudal	rostral	0.105186344	0.1351623	0.863328222	-0.274869263	0.485241952
AuD	caudal	ventral	0.054153759	0.128423944	0.974075958	-0.306954627	0.415262146
AuD	core	rostral	0.014269375	0.14599197	0.999653026	-0.396237599	0.424776348
AuD	core	ventral	-0.036763211	0.13977667	0.993410924	-0.429793715	0.356267294
AuD	rostral	ventral	-0.051032585	0.13977667	0.982852285	-0.44406309	0.341997919
AuV	caudal	core	-0.178962009	0.059633799	0.033957627	-0.346643082	-0.011280935
AuV	caudal	rostral	-0.012922918	0.059633799	0.996279102	-0.180603992	0.154758155
AuV	caudal	ventral	-0.176507454	0.056660827	0.026758077	-0.335828987	-0.017185922
AuV	core	rostral	0.166039091	0.064411864	0.079366954	-0.015077169	0.34715535
AuV	core	ventral	0.002454554	0.061669665	0.999976489	-0.170951062	0.175860171
AuV	rostral	ventral	-0.163584536	0.061669665	0.068623354	-0.336990153	0.00982108
Insura	caudal	core	-0.0443271	0.072841263	0.928105504	-0.249145532	0.160491331
Insura	caudal	rostral	-0.08182911	0.072841263	0.680019329	-0.286647542	0.122989321
Insura	caudal	ventral	-0.107241499	0.069209848	0.429380769	-0.301848951	0.087365953
Insura	core	rostral	-0.03750201	0.078677556	0.963329713	-0.2587312	0.183727181
Insura	core	ventral	-0.062914398	0.075328025	0.837103125	-0.274725223	0.148896426
Insura	rostral	ventral	-0.025412389	0.075328025	0.986359001	-0.237223213	0.186398436
S1/S2	caudal	core	-0.036567355	0.057248758	0.9181393	-0.19754206	0.12440735
S1/S2	caudal	rostral	-0.019817321	0.057248758	0.985300598	-0.180792026	0.141157384
S1/S2	caudal	ventral	0.042264783	0.054394689	0.863867142	-0.110684719	0.195214285
S1/S2	core	rostral	0.016750034	0.061835726	0.992814897	-0.15712252	0.190622587
S1/S2	core	ventral	0.078832138	0.0592032	0.55511636	-0.087638158	0.245302434
S1/S2	rostral	ventral	0.062082104	0.0592032	0.72366849	-0.104388192	0.2285524
V1/V2	caudal	core	-0.017190984	0.084378776	0.99690067	-0.254451129	0.220069162
V1/V2	caudal	rostral	-0.201793064	0.084378776	0.112811288	-0.43905321	0.035467081
V1/V2	caudal	ventral	-0.051144586	0.080172173	0.918415924	-0.276576409	0.174287236
V1/V2	core	rostral	-0.18460208	0.091139495	0.213739242	-0.440872327	0.071668167
V1/V2	core	ventral	-0.033953602	0.087259423	0.979401241	-0.279313684	0.211406479
V1/V2	rostral	ventral	0.150648478	0.087259423	0.338103334	-0.094711603	0.396008559

Table S13, related to Figure 8D. Statistical parameters.

Input nucleus	Difference	StdErr	pValue	Lower	Upper
Au1	0.374503419	0.087262561	0.003603234	0.168160252	0.580846586
AuD	-0.135257895	0.221836357	0.561309537	-0.659817525	0.389301736
AuV	0.225220702	0.049675215	0.002687303	0.107757484	0.34268392
Insura	0.063749362	0.046614926	0.213732353	-0.046477422	0.173976146
S1/S2	0.103115713	0.018469983	0.000830732	0.059441144	0.146790282
V1/V2	0.040261081	0.024590069	0.145581585	-0.017885192	0.098407354

Table S14, related to Figure 8E. Statistical parameters.

Input nucleus	group 1	group 2	Difference	StdErr	pValue	Lower	Upper
Au1	caudal	core	-0.001283979	0.02723212	0.999960927	-0.077856514	0.075288556
Au1	caudal	rostral	-0.026176731	0.02723212	0.772556818	-0.102749265	0.050395804
Au1	caudal	ventral	-0.026465041	0.025874495	0.738384693	-0.099220143	0.046290061
Au1	core	rostral	-0.024892752	0.029414051	0.831770858	-0.107600542	0.057815039
Au1	core	ventral	-0.025181062	0.02816181	0.807936423	-0.104367743	0.054005618
Au1	rostral	ventral	-0.000288311	0.02816181	0.9999996	-0.079474991	0.07889837
AuD	caudal	core	0.085200992	0.068919277	0.612428228	-0.108589418	0.278991401
AuD	caudal	rostral	0.047041754	0.068919277	0.902444833	-0.146748655	0.240832164
AuD	caudal	ventral	0.029403418	0.065483388	0.969012692	-0.1547258	0.213532637
AuD	core	rostral	-0.038159237	0.074441327	0.955065533	-0.247476803	0.171158328
AuD	core	ventral	-0.055797573	0.071272144	0.861262387	-0.256203885	0.144608738
AuD	rostral	ventral	-0.017638336	0.071272144	0.994492132	-0.218044648	0.182767976
AuV	caudal	core	0.096677592	0.085728361	0.67745407	-0.144377379	0.337732563
AuV	caudal	rostral	0.074639879	0.085728361	0.819796153	-0.166415092	0.31569485
AuV	caudal	ventral	0.057592706	0.081454476	0.893050143	-0.171444755	0.286630168
AuV	core	rostral	-0.022037714	0.092597213	0.995091633	-0.28240684	0.238331413
AuV	core	ventral	-0.039084886	0.088655082	0.970580817	-0.288369346	0.210199574
AuV	rostral	ventral	-0.017047172	0.088655082	0.997389271	-0.266331632	0.232237288
Insura	caudal	core	-0.002902991	0.012631249	0.995572645	-0.03842012	0.032614139
Insura	caudal	rostral	-0.011177715	0.012631249	0.812630077	-0.046694845	0.024339414
Insura	caudal	ventral	-0.016327425	0.012001533	0.537892699	-0.05007389	0.01741904
Insura	core	rostral	-0.008274725	0.013643308	0.92875318	-0.046637609	0.03008816
Insura	core	ventral	-0.013424435	0.013062473	0.735614117	-0.0501541	0.023305231
Insura	rostral	ventral	-0.00514971	0.013062473	0.978612919	-0.041879375	0.031579956
S1/S2	caudal	core	0.191194302	0.124057932	0.433964124	-0.15763753	0.540026134
S1/S2	caudal	rostral	0.177146423	0.124057932	0.498186758	-0.171685409	0.525978255
S1/S2	caudal	ventral	0.164645894	0.117873171	0.516395122	-0.166795342	0.496087129
S1/S2	core	rostral	-0.014047879	0.133997882	0.999572105	-0.390829321	0.362733562
S1/S2	core	ventral	-0.026548408	0.128293204	0.996754187	-0.387289174	0.334192358
S1/S2	rostral	ventral	-0.012500529	0.128293204	0.99965624	-0.373241295	0.348240237
V1/V2	caudal	core	0.04272312	0.062161145	0.900663552	-0.132064462	0.217510702
V1/V2	caudal	rostral	0.010530157	0.062161145	0.9982085	-0.164257425	0.185317738
V1/V2	caudal	ventral	-0.03924511	0.059062175	0.909100061	-0.205318864	0.126828645
V1/V2	core	rostral	-0.032192963	0.06714171	0.962716562	-0.220985129	0.156599203
V1/V2	core	ventral	-0.08196823	0.064283293	0.589005142	-0.262722967	0.098786508
V1/V2	rostral	ventral	-0.049775266	0.064283293	0.865051867	-0.230530004	0.130979471

Table S15, related to Figure 8F. Statistical parameters.

Input nucleus	Difference	StdErr	pValue	Lower	Upper
Au1	0.013036116	0.017199721	0.473234133	-0.027634761	0.053706992
AuD	-0.122800814	0.109801392	0.300306571	-0.382439849	0.13683822
AuV	-0.154796492	0.146443113	0.325601299	-0.501079429	0.191486444
Insura	0.007339548	0.00645818	0.29316346	-0.007931621	0.022610717
S1/S2	-0.313741872	0.207936273	0.17507976	-0.805433025	0.177949282
V1/V2	-0.008886787	0.008485602	0.329779854	-0.028952047	0.011178472

Table S16, related to Figure 8G. Correlation coefficients between every combination of starter nuclei and input layers.

	Layer2-3	Layer4	Layer5	Layer6
BIC	-0.0403	0.061733	0.318674	-0.29476
DLG	0.09567	0.088658	0.19437	-0.20922
Eth/REth	-0.0746	-0.1623	-0.02127	0.022104
IGL/PrG	0.223998	0.374774	0.027005	-0.01538
LP/LD	0.151269	0.203361	-0.06012	0.016886
MGV	0.008296	-0.02338	-0.23326	0.214437
MGD	0.327899	0.338491	-0.18185	0.152033
MGM/SG	0.153057	0.126801	0.0899	-0.09677
PP/PIL	-0.0878	-0.05702	0.253256	-0.23183
Po	0.02621	-0.05419	-0.22305	0.202982
SN/VTA	0.015339	-0.22241	0.225119	-0.18357
ZI	-0.19126	-0.18701	0.127831	-0.12231
subB	-0.1704	-0.15287	-0.02844	0.058718
mRt/p1Rt	0.237748	0.049424	-0.11957	0.11958

Table S17, related to Figure 8. P-values of correlation coefficients between every combination of starter nuclei and input layers.

	Layer2-3	Layer4	Layer5	Layer6
BIC	0.954092	0.954092	0.866904	0.866904
DLG	0.954092	0.954092	0.866904	0.866904
Eth/REth	0.954092	0.866904	0.954092	0.954092
IGL/PrG	0.866904	0.866904	0.954092	0.954092
LP/LD	0.866904	0.866904	0.954092	0.954092
MGV	0.965932	0.954092	0.866904	0.866904
MGD	0.866904	0.866904	0.866904	0.866904
MGM/SG	0.866904	0.910799	0.954092	0.954092
PP/PIL	0.954092	0.954092	0.866904	0.866904
Po	0.954092	0.954092	0.866904	0.866904
SN/VTA	0.954092	0.866904	0.866904	0.866904
ZI	0.866904	0.866904	0.910799	0.910799
subB	0.866904	0.866904	0.954092	0.954092
mRt/p1Rt	0.866904	0.954092	0.910799	0.910799
